# Reusing spent graphite through direct electropolymerisation of polypyrrole for sustainable pseudocapacitive electrodes

**DOI:** 10.1039/d6ra05302e

**Published:** 2026-08-20

**Authors:** Nishat S. Khan, Maryna Taryba, R. G. Duarte, M. F. Montemor, R. S. Sampaio

**Affiliations:** a CQE – Centro de Química Estrutural, IMS – Institute of Molecular Sciences, Departamento de Engenharia Química, Instituto Superior Técnico, Universidade de Lisboa Av. Rovisco Pais 1049-001 Lisboa Portugal nishat.khan@tecnico.ulisboa.pt; b Instituto Politécnico de Setúbal, EST Barreiro 2839-001 Lavradio Portugal

## Abstract

Lithium-ion battery recycling is largely focused on metal recovery, while graphite-rich residues remain comparatively undervalued despite their critical-material relevance and intrinsic conductivity. Here, spent graphite recovered from LIB waste was directly upgraded into a pseudocapacitive electrode *via* polypyrrole electropolymerisation, thereby avoiding high-temperature treatment, complex chemical activation, and expensive active materials. PPy was deposited onto spent graphite from 0.1 M pyrrole solution, producing cauliflower-like conductive coatings with improved electrochemical activity. The electrodes were evaluated by cyclic voltammetry, galvanostatic charge–discharge, and electrochemical impedance spectroscopy in 1 M H_2_SO_4_, demonstrating good electrochemical performance. A symmetric cell was constructed, achieving a specific capacitance of 37.8 F g^−1^ at 0.5 A g^−1^, with excellent rate capability and 86.3% capacitance retention after 5000 cycles. This work demonstrates a simple and scalable route for converting graphite-rich battery waste into sustainable pseudocapacitive electrodes for high-power energy-storage applications.

## Abstract 2

Spent LIB graphite is directly upgraded by PPy electropolymerisation into a pseudocapacitive electrode, providing a simple route for valorising battery waste for high-power energy storage.

## Graphical abstract

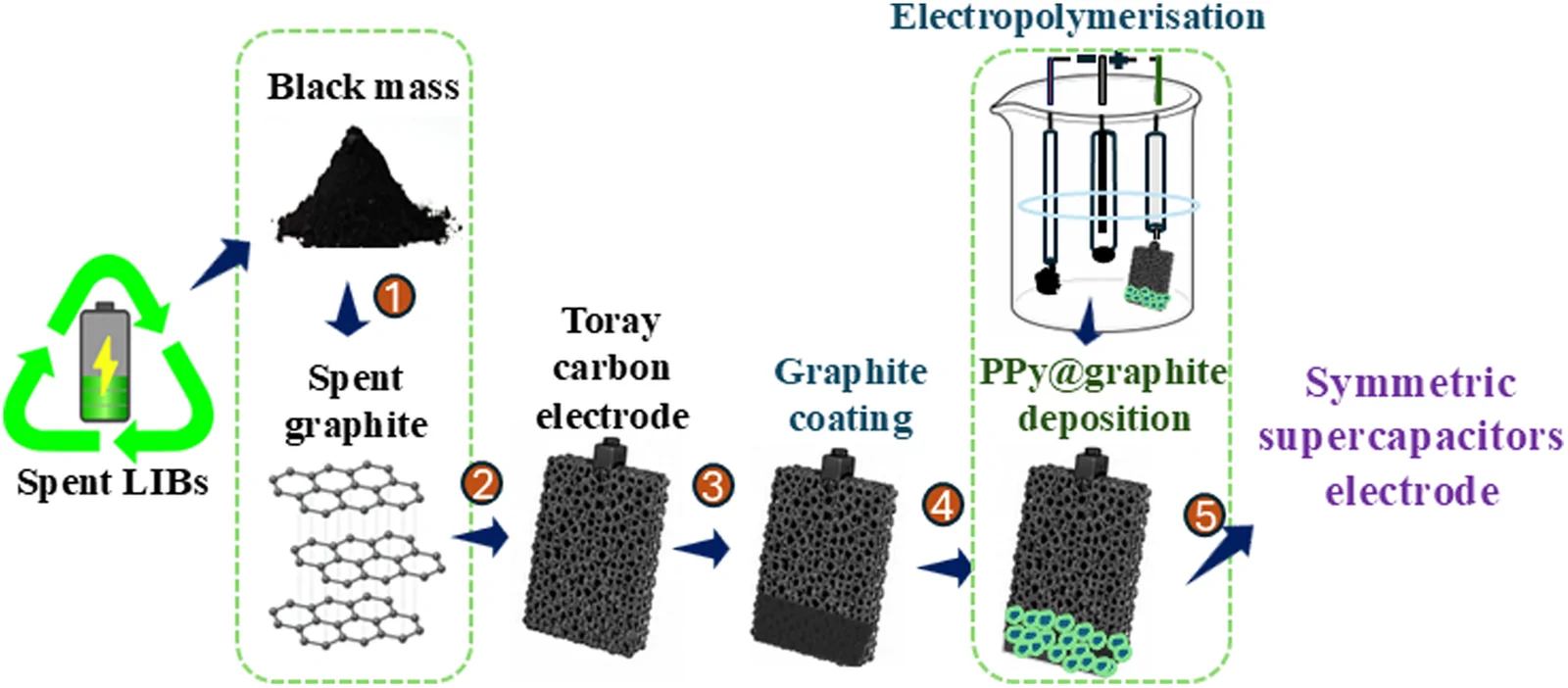

## 1. Introduction

The use of lithium-ion batteries (LIBs) has increased significantly across multiple sectors, including electric vehicles and portable electronics, in recent years.^1^ Consequently, the accumulation of end-of-life batteries has intensified, creating challenges for their effective management. Industrial recycling processes primarily focus on recovering valuable metals, while the graphite fraction is often overlooked and discarded as a low-value byproduct.^2–4^ Graphite is the dominant commercial anode material in LIBs owing to its high electrical conductivity, chemical stability, and favourable lithium-intercalation properties.^5^ Despite prolonged electrochemical cycling and recycling treatments, spent graphite largely preserves its graphitic framework and electrical conductivity and facilitates electron transfer, enabling its suitability for reuse in various applications, including energy storage devices such as supercapacitors.^6^ In addition, structural defects in recycled carbon materials can enhance electrochemical activity by increasing the number of active sites and facilitating charge transfer compared to commercial graphite. This material offers a more sustainable and cost-effective alternative by avoiding additional purification and processing steps. Although the electrochemical performance of recovered graphite is generally inferior to that of commercial graphite, it can be significantly improved through composite design and surface functionalisation.^7^ It can be enhanced by developing advanced graphite-based composites for supercapacitor applications. Supercapacitors, also known as electrochemical capacitors, have attracted considerable attention as advanced energy storage devices because of their high power density, rapid charge–discharge capability, and excellent cycling stability. Based on their charge storage mechanisms, supercapacitors are generally classified into three categories: electrical double-layer capacitors (EDLCs), pseudocapacitors, and hybrid supercapacitors. EDLCs store energy by electrostatically accumulating electrolyte ions at the electrode/electrolyte interface, without involving faradaic reactions. Carbon-based materials such as activated carbon, graphene, carbon nanotubes, and graphite are commonly employed as EDLC electrodes because of their high surface area and excellent electrical conductivity.^8^ In contrast, pseudocapacitors store charge through fast and reversible faradaic redox reactions occurring at or near the electrode surface. Typical pseudocapacitive materials include conducting polymers such as polypyrrole (PPy), polyaniline (PANI), and poly(3,4-ethylenedioxythiophene) (PEDOT), as well as transition metal oxides and hydroxides, including MnO_2_, RuO_2_, and Ni(OH)_2_.^9,10^ Hybrid supercapacitors combine EDLC-type carbon materials with pseudocapacitive components to exploit the advantages of both mechanisms, thereby achieving improved energy density while maintaining high power density and long cycling life.^11,12^

Consequently, hybrid electrode architectures have emerged as an effective strategy for developing high-performance supercapacitors. From this perspective, conducting polymers can be incorporated onto spent graphite to create novel electroactive materials for supercapacitor electrodes. Conductive polymers are inexpensive and relatively easy to prepare and exhibit high electrical conductivity.^13^ Conductive polymers such as polyaniline (PANI),^14^ polythiophene,^15^ polycarbazole,^16^ and polypyrrole (PPy),^17^ have attracted significant interest in the scientific community. Conducting polymers used in supercapacitor electrodes typically exhibit limited cycle stability under prolonged charge–discharge cycles. To address this limitation, composites combining conductive polymers and carbon materials have been proposed, exhibiting superior electrochemical properties compared to individual materials.^18,19^

Conducting polymers can be synthesised through chemical or electrochemical methods. Electrochemical polymerisation is an attractive route for fabricating PPy films.^20^ This approach involves the oxidation of the pyrrole monomer, dissolved in a solvent containing an ionic dopant, at the electrode surface under an anodic potential.^21^ The pyrrole monomer is oxidised at the electrode surface to form reactive radicals, which couple at their α-positions to form α,α′-dimers with proton loss and rearomatisation. These dimers further react with additional radicals, leading to chain propagation and film formation.^22^ The growth transient curve reveals distinct stages: a sharp current drop due to double-layer formation and monomer oxidation, followed by a current minimum as insoluble oligomers begin to deposit. As polymer nucleation progresses, the current increases until it reaches a steady plateau, reflecting a more compact inner layer and diffusion limitations of doping ions.^23,24^ Higher applied potentials accelerate both double-layer formation and nucleation, resulting in faster film growth.

A major challenge in PPy electropolymerisation is achieving adherent coatings on reactive metals, which may oxidise before polymer formation due to their lower oxidation potentials. As a result, electropolymerisation is commonly performed on conductive and electrochemically stable substrates to minimise substrate oxidation or dissolution.

Several studies have reported graphite/PPy and graphene/PPy composites for supercapacitor electrodes. C. J. Raj *et al.* developed a graphite/graphene oxide/PPy electrode, which showed an areal capacitance of 1320 mF cm^−2^ along with 83% capacitance retention.^25^ Hao-Hsiang Chang successfully developed RGO/PPy, achieving a specific capacitance of 424 F g^−1^.^26^ Additionally, Haihan Zhou fabricated PPy/graphene oxide, showing an areal capacitance of 152 mF cm^−2^.^27^ However, these systems commonly rely on commercial carbon materials, graphene oxide/reduced graphene oxide, electrochemical exfoliation, chemical reduction, or multistep electrode fabrication. In contrast, the present work directly employs spent graphite recovered from lithium-ion battery waste as the conductive scaffold for PPy electropolymerisation. This work differs from our previous MnOx-based modification of spent graphite.^28^ It introduces a conducting-polymer route to enhance pseudocapacitive charge storage *via* a simple, low-energy process. Polypyrrole (PPy) was selected in this study because it is one of the most widely investigated conducting polymers for supercapacitor electrodes owing to its high electrical conductivity, fast and reversible redox activity, ease of electropolymerization, and relatively low cost.^29^ Although numerous studies have reported PPy-based electrodes and various strategies to improve their electrochemical performance, the use of recovered spent graphite as a conductive scaffold and active support for electrodeposited PPy remains comparatively underexplored. Recovered spent graphite provides excellent electrical conductivity, a layered carbon structure, and an environmentally sustainable alternative to virgin graphite, while electrodeposited PPy introduces additional pseudocapacitance through reversible redox reactions.^30^ The combination of these materials is expected to enhance the overall capacitance and charge-transfer characteristics while promoting the valorisation of waste graphite from spent batteries.^31^ Therefore, this work extends existing PPy-based supercapacitor research by integrating electrodeposited PPy with recovered spent graphite to develop a high-performance and sustainable electrode material. The influence of deposition time and growth potential on morphology, electrochemical behaviour, impedance response, and cycling stability was systematically investigated. The novelty of this work lies in the direct use of hydrometallurgically recovered spent graphite as both a conductive scaffold and an electrode substrate for PPy electropolymerisation, avoiding graphene conversion, high-temperature activation, and multistep carbon processing. This strategy provides a mild and scalable route to transform an undervalued, graphite-rich recycling residue into a sustainable pseudocapacitive electrode material.

## 2. Experimental

### 2.1. Materials

Pyrrole (C_4_H_5_N, > 98%) and lithium perchlorate (LiClO_4_) were acquired from Alfa Aesar. Black mass was purchased from NoCanary. Commercial sulfuric acid (H_2_SO_4_, 95–98%, Carl Roth) and nitric acid (HNO_3_, 68–70%, Fisher) were used. The growth and characterisation solutions were prepared using Millipore water with a resistivity of 15 MΩ cm at 25 °C.

### 2.2. Electrode fabrication

Initially, the commercial black mass was leached with concentrated HNO_3_ for 48 h at room temperature using a solid-to-liquid ratio of 1 g of black mass per 100 mL of acid. The resulting residue was then thoroughly washed with water until a neutral pH was reached and dried for 24 h. This treatment followed a previously reported procedure to remove impurities, such as residual metals and organic compounds, and to enhance its wettability.^28^ As reported, the initial black mass contains significant amounts of Ni, Mn, and Co, indicating that it originates from a mixture of cathode and anode materials from spent NMC batteries. After leaching, the composition is predominantly carbon, with only small amounts of residual metals remaining. The morphology and electrochemical behaviour of the recovered graphite are shown in Fig. S1 and S2, respectively.

The deposition of spent graphite onto Toray sheet was achieved using an ink composed of 95% active material and 5% polyvinylidene fluoride (PVDF) as a binder, dispersed in *N*-methyl-2-pyrrolidone (NMP, >99.8%). The working electrode was a 1 cm^2^ area (0.5 cm^2^ on each side of the Toray substrate). The mass loadings were controlled using a Sartorius MC5-0CE microbalance with an accuracy of 0.01 mg. These spent graphite electrodes were dried in an oven at 50 °C for 12 hours.

Then, a polypyrrole film was electrochemically synthesised onto the graphite layer by chronoamperometry in a solution of 0.1 M pyrrole and 0.1 M LiClO_4_ as the supporting electrolyte. The electropolymerisation parameters, namely, time and growth potential, were studied and correlated with the electrochemical performance of the composite. The pristine graphite electrode exhibited a specific capacitance of only 0.43 F g^−1^ (Fig. S2), confirming the essential role of PPy in enhancing electrochemical performance. The PPy loading was determined gravimetrically from the mass difference of the electrodes before and after electropolymerisation, as summarised in Table S1. After composite synthesis, the resulting electrodes were rinsed with abundant water to remove residual solution and dried at 50 °C for 24 hours. Two sets of composites were produced. One was designated as GPT_09, GPT_16, GPT_25, and GPT_36, corresponding to different times of electropolymerisation at a constant potential of 0.70 V *vs.* SCE for 9, 16, 25, and 36 minutes, respectively. The electrodes in the other set were obtained with a constant pulse time of 16 minutes and were designated as GPV_0.65, GPV_0.75, GPV_0.80, and GPV_0.85, corresponding to the application of different potentials of electropolymerisation: 0.65, 0.75, 0.80 and 0.85 V *vs.* SCE, respectively. Table 1 summarises the experimental parameters used for the potentiostatic synthesis of the electrodes.

**Table 1:** Experimental parameters used for the potentiostatic synthesis of the electrodes

Sample	*E*_g_ *vs.* SCE (V)	Time (min)
GPT_09	0.70	9
GPT_16	0.70	16
GPT_25	0.70	25
GPT_36	0.70	36
GPV_0.65	0.65	16
GPV_0.75	0.75	16
GPV_0.80	0.80	16
GPV_0.85	0.85	16

### 2.3. Electrochemical setup

A three-electrode configuration consisted of a platinum wire as a counter electrode, a saturated calomel electrode (SCE) (Hanna Instruments) as a reference, and the prepared composite material serving as the working electrode. This configuration was used to perform the electrochemical synthesis and characterisation of the electrodes. For the symmetric device characterisation, a two-electrode configuration was assembled. All electrochemical measurements were conducted using a potentiostat (Gamry 5000E) in a Faraday cage at room temperature. The growth solution was deaerated with nitrogen for 20 min before electrodeposition to remove dissolved oxygen.

### 2.4. Electrochemical characterisation

The electrochemical response of the different electrodes was assessed using cyclic voltammetry and galvanostatic charge–discharge (GCD) tests in an aqueous 1 M H_2_SO_4_ solution. The CV was conducted at scan rates from 2 to 200 mV s^−1^, and GCD tests were executed at the current densities from 0.5 to 10.0 A g^−1^. The electrodes' stability was evaluated by GCD for 5000 cycles at 10 A g^−1^, within the optimum working potential window. The relevant metrics, such as specific capacitance, ohmic drop, and coulombic efficiency, were calculated using the 3rd cycle of GCD or CV tests.

Electrochemical impedance spectroscopy (EIS) measurements were carried out applying a 10 mV rms potential perturbation around the open-circuit potential value, in the frequency range from 100 kHz to 10 mHz, and with 10 points per decade distributed logarithmically. EIS data fitting was achieved with the ZView® program (Scribner Associates, Southern Pines, NC, USA).

In the EIS fitting procedure, capacitors were replaced with constant phase elements (CPEs) due to non-ideal behaviour. The impedance of a CPE is given by the following eqn (1):^32^1
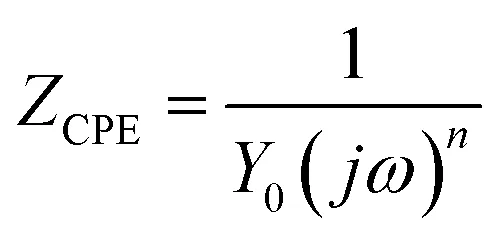
where *Y*_0_ is the frequency-independent admittance, *n* is the power of the CPE, *ω* is the angular frequency, and 
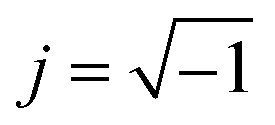
. The areal capacitance (*C*_A_) of the modified electrodes was estimated through the fitting parameters according to the Brug eqn (2):^33^2
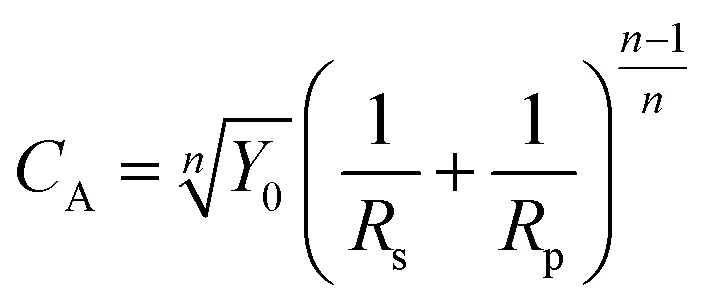
where *R*_s_ and *R*_p_ are the resistances in series and in parallel, respectively, with the corresponding CPE.

### 2.5. Physicochemical characterisation of the electrodes

Particle morphologies were examined using scanning electron microscopy (SEM) with a Thermo Scientific PhenomTM ProX G6 SEM instrument. The crystalline structure was analysed by a Bruker D8 Advance powder diffractometer with a Linxeye-XE detector (Cu Kα, *λ* = 1.5406 Å). Raman spectroscopy was carried out on a Horiba LabRAM HR800 Evolution spectrometer, equipped with a solid-state laser operating at 532 nm focused with a 50× objective lens. Spectra were collected in the range of 400 and 2000 cm^−1^ in four accumulations and an acquisition time of 10 s, using a spectrograph with 600 lines per mm grating.

## 3. Results and discussion

### 3.1. Electrochemical deposition

**Fig. 1:**
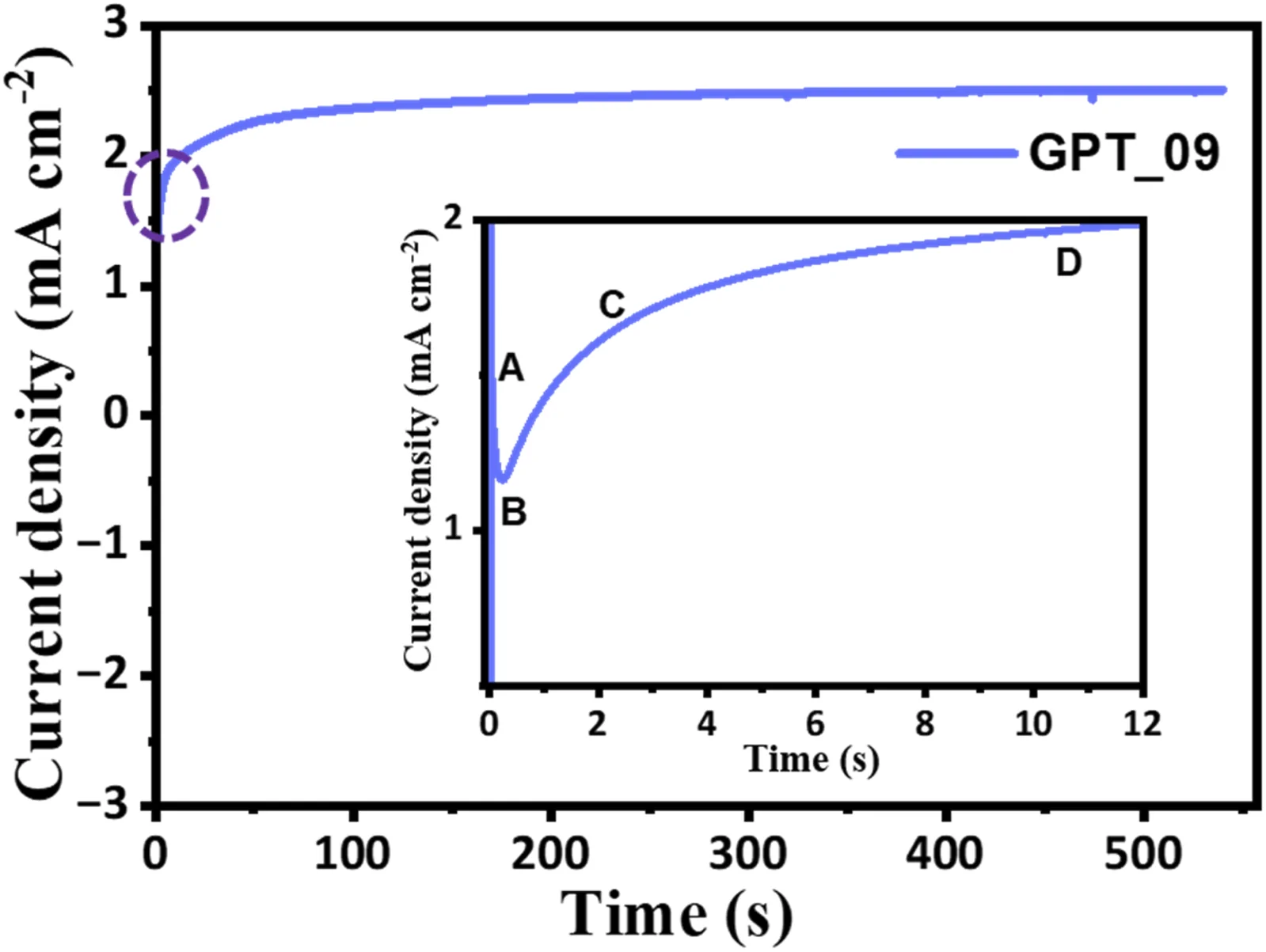
Time–current transient of the electropolymerisation of polypyrrole at 0.70 V *vs.* SCE for 9 min (inset of growth film highlighted in a purple circle).

The deposition of polypyrrole was carried out electrochemically through the application of an anodic potential pulse. The potential range for conducting polymer electrochemical growth was initially determined through cyclic voltammetry to identify the minimum potential value for monomer oxidation and the maximum to avoid polymer overoxidation. The potentiostatic synthesis of polypyrrole at 0.70 V *vs.* SCE for 9 minutes is depicted in Fig. 1. The total charge density of growth was 1.3 C cm^−2^. The PPy loading was determined from the mass difference of the graphite electrodes before and after electropolymerisation using a high-precision microbalance. The measured mass increase was consistent with the charge passed during electropolymerisation, confirming the successful deposition of PPy. The masses of the electrodes are shown in Table S1.

The inset in Fig. 1 presents the different stages of the electropolymerisation process. Initially, the current density drops abruptly due to the oxidation of the monomers, the subsequent formation of oligomers, and the formation of the double layer (*A*).^34^ When the oligomers reach a specific size that makes them insoluble in the solution, they begin to precipitate on the substrate surface, leading to the formation of the first polymer nuclei, and the current density reaches a minimum value (*B*). Then, the current begins to increase gradually, as the nucleation process progresses, until the substrate surface gets fully covered by nuclei (*C*). Finally, the current density reaches a plateau and increases slightly as the film thickness increases, which hinders the diffusion of doping ions into the polymeric matrix and increases its resistivity (*D*).^35^

### 3.2. Morphological analysis

**Fig. 2:**
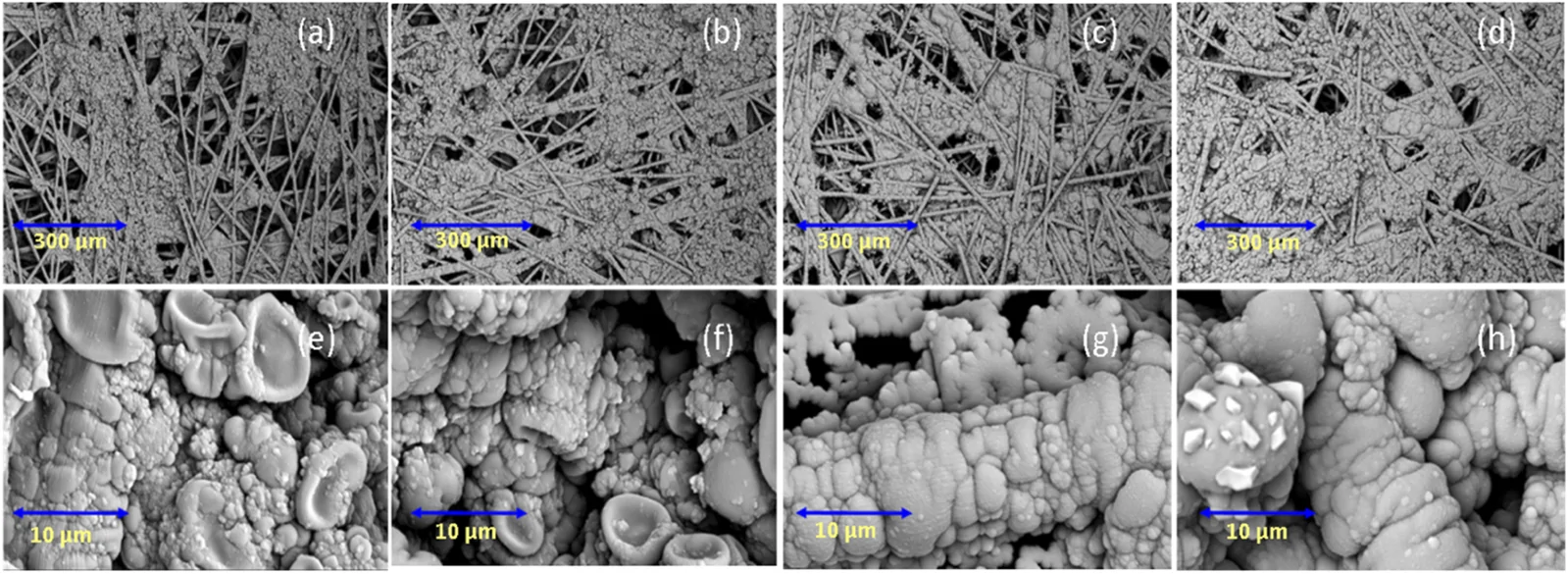
SEM images of the polypyrrole-graphite composite electrodes grown at 0.70 V for varying durations at (a and e) 09 min, (b and f) 16 min, (c and g) 25 min, and (d and h) 36 min.

The morphology and surface characteristics of GPT and GPV samples were examined by scanning electron microscopy (SEM). Fig. 2 illustrates the morphological features of the PPy composites obtained for different times of electrochemical polymerisation (09, 16, 25, and 36 minutes). Fig. 2(a–h) present GPT_09, GPT_16, GPT_25, and GPT_36. During shorter time pulses, the polymer appears as scattered granules. However, longer deposition times result in more uniform coverage and the development of granular, irregular aggregates that display an interconnected network across the graphite surface. This configuration creates a more interconnected and roughened surface morphology, being advantageous for energy-storage electrode applications. Increasing deposition times led to a gradual increase in the PPy particle size and also in their agglomeration. Initially, incipient globular particles were detected, which became denser and underwent closer packing through prolonged electropolymerisation. A compact and unevenly organised film was obtained in 36 minutes. The SEM observations highlight that the deposition time strongly affects the morphology of the resulting composite material.

**Fig. 3:**
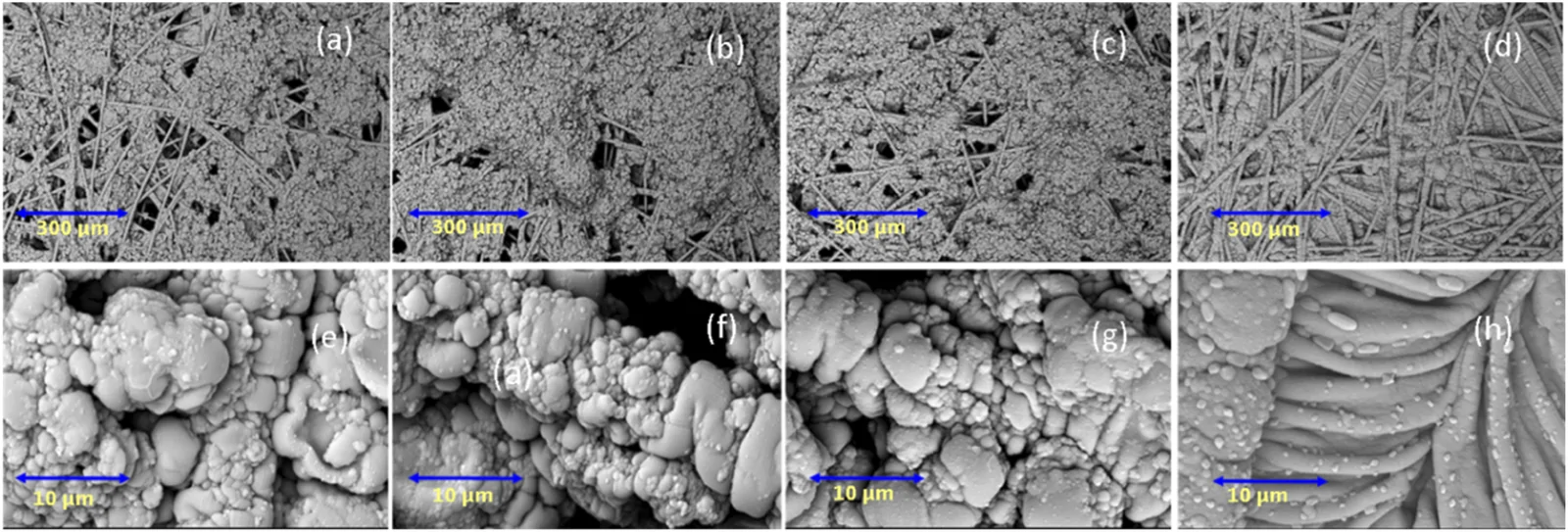
SEM images of PPy-graphite composite electrodes at a constant deposition time of 16 minutes under varying applied potentials: (a and e) 0.65 V, (b and f) 0.75 V, (c and g) 0.80 V, and (d and h) 0.85 V.

Fig. 3(a–h) present GPV_0.65, GPV_0.75, GPV_0.80, and GPV_0.85. These micrographs illustrate the transformation of PPy onto spent graphite as the electrochemical polymerisation potential increases to 0.65, 0.75, 0.80, and 0.85, respectively. The resulting material exhibits a cauliflower-like structure, in agreement with the literature.^36^ The higher potential facilitates uniform coverage of the spent graphite within a reduced timeframe. At lower potentials (0.65 V), the electrode surface shows limited coverage due to slower PPy nucleation. As the potential increases, nucleation occurs rapidly, resulting in a denser, more agglomerated PPy film, which increases the electrode's surface area. The composite produced at 0.85 V exhibits the most extensive surface coverage compared to the other applied potentials, consistent with the higher electropolymerisation rate at elevated potentials.^37^

SEM-EDS analysis was performed to investigate the elemental composition of the PPy-coated graphite electrodes, and the results are summarised in Table S2. Carbon is the predominant element in all samples, consistent with the graphitic substrate, while the presence of nitrogen (7.5–10.1 wt%) confirms the successful electropolymerization of polypyrrole on the graphite surface. Oxygen and chlorine are mainly associated with perchlorate counter-ions and surface oxygen-containing species. Small amounts of aluminium together with trace quantities of Mn, Co, and Ni (≤0.5 wt%) are observed and are attributed to residual impurities originating from the recycled graphite precursor.^38^ The low concentrations of these transition metals indicate that they are unlikely to contribute significantly to the electrochemical response. Therefore, the pseudocapacitive behaviour is primarily associated with the reversible redox (doping/dedoping) reactions of the PPy coating, while the recovered graphite acts as the conductive framework.^25,29^

### 3.3. Structural analysis

The crystallographic structure and the phase information of the synthesised electrodes were investigated by XRD and Raman spectroscopy, respectively. The XRD pattern of the recovered graphite before electropolymerisation is presented in Fig. S3. The diffractogram is dominated by the characteristic graphite (002) reflection at 2*θ* ≈ 26.6°, indicating preservation of the crystalline graphitic structure. A low-intensity reflection near 18° and 45° is attributed to a residual layered cathode-derived phase.^38^

**Fig. 4:**
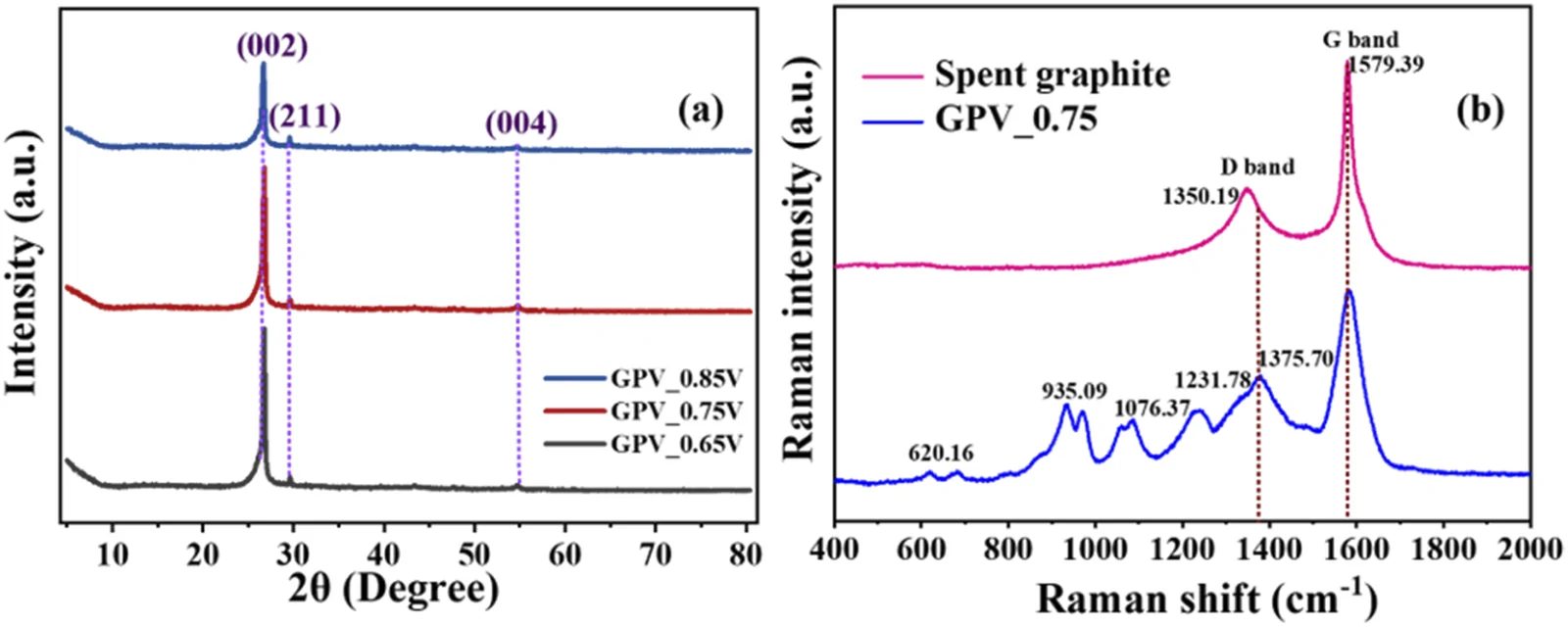
(a) XRD diffractograms of GPV-0.65, GPV-0.75, GPV-0.85, and (b) Raman spectra of GPV-0.75 and spent graphite.

After electropolymerisation, Fig. 4(a) shows the XRD patterns of GPV_0.65 V, GPV_0.75 V, and GPV_0.85 V. The XRD diffractograms of the PPy-graphite composites at different deposition potentials display characteristic peaks at 2*θ* values of 26.64°, 29.64°, and 54.06°. The sharp diffraction peak at 26.64° corresponds to the (002) plane of crystalline graphite, reflecting the layered graphitic structure with an interlayer spacing of 0.336 nm.^39,40^ The peak at 29.64° is assigned to the short-range ordering of PPy chains, which arises from the periodic arrangement of the polymer backbone and dopant-induced structural reorganisation during electropolymerisation.^41^ This feature is relatively broad, consistent with the largely amorphous nature of polypyrrole. The diffraction peak at 54.06° can be indexed to the (004) plane of graphite, further confirming the crystallinity of the underlying substrate.^24^ However, variations in the electropolymerisation potential influence the relative intensities of these peaks: at lower potentials, the graphite reflections dominate due to thinner PPy coverage, whereas at higher potentials, the characteristic broad PPy peak becomes more prominent as thicker films with improved structural ordering form.

Fig. 4(b) shows the Raman spectra of graphite, which display two dominant bands: the D-band at 1350.19 cm^−1^, which arises from the breathing mode of sp^2^ carbon atoms associated with structural disorder such as edge defects and lattice distortions, and the G-band at 1579.39 cm^−1^, corresponding to the in-plane vibration of sp^2^ bonded carbon atoms in the graphitic lattice. Upon electropolymerisation of PPy on graphite at different potentials, additional Raman features emerge alongside the graphite peaks, confirming the successful polymer deposition. The peaks at 620.16 cm^−1^ and 935.09 cm^−1^ can be attributed to the C–C and C–H in-plane deformation modes of the pyrrole ring, while the peak at 1076.37 cm^−1^ corresponds to the C–H in-plane deformation vibration. The band at 1231.78 cm^−1^ is associated with the C–N stretching vibration of the pyrrole ring.^42,43^ The presence of both graphite-related (1375.70 cm^−1^ and 1579.39 cm^−1^) and PPy-related peaks indicates the coexistence of the carbon substrate and the polymer layer,^44^ and variations in peak intensities at different electropolymerisation potentials reflect changes in polymer growth, structural ordering, and the degree of interaction between polypyrrole chains and the graphite substrate. The apparent peak-intensity ratio, *I*_D_/*I*_G_, calculated from the maximum intensities of the D and G bands, increased from 0.27 for the recovered graphite to 0.35 for GPV_0.75. This modest increase is consistent with additional disorder-related and PPy-derived vibrational contributions introduced during electropolymerisation and interfacial interaction with the graphite surface.^29^ Together with the retention of the characteristic graphite reflections in the XRD patterns, the results point to surface functionalisation rather than substantial disruption of the graphitic framework.

### 3.4. Selection of the test electrolyte

The electrochemical response of the GPT_09 electrode was investigated in different electrolytic solutions, specifically 1 M H_2_SO_4_, 1 M Na_2_SO_4_, and 1 M KOH, to study the effect of the pH on the performance of the composite in a 3-electrode configuration.

The specific capacitance (*C*_s_) was calculated from the GCD curves, according to the mathematical eqn (3):3
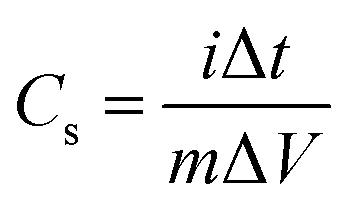
where *i* is the discharge current (*A*), Δ*t* is the discharge time (s), *m* is the mass of active material (g), and Δ*V* is the potential window (V) (Δ*V* = *V*_2_ − *V*_1_). The ohmic drop was determined by the potential difference between the last point of the charging and the first point of the discharging transients, respectively. In cyclic voltammograms, *C*_s_ was determined by eqn (4):4
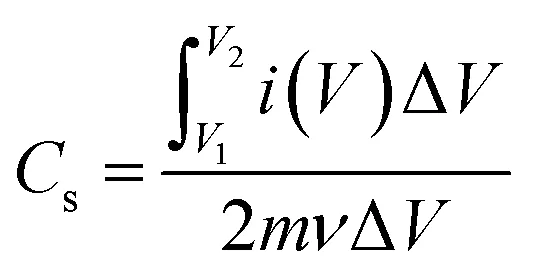
where *ν* represents the scan rate, and *V*_2_ and *V*_1_ signify the anodic and cathodic potential limits, respectively.

**Fig. 5:**
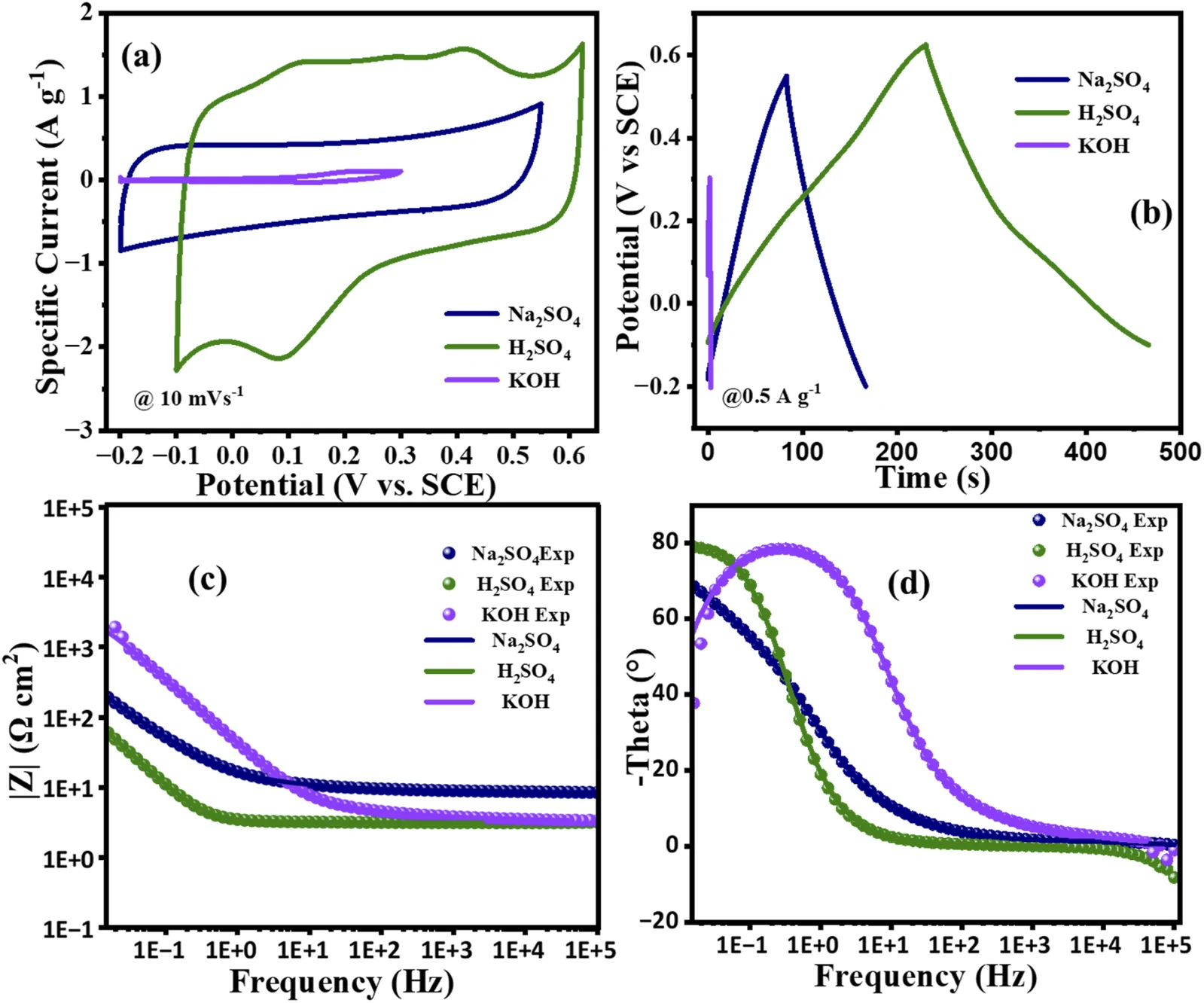
Effect of the electrolyte on the GPT-09 electrochemical performance: (a) cyclic voltammetry, (b) galvanostatic charge/discharge, (c) magnitude of the impedance, (d) phase angle.

Fig. 5(a and b) presents the cyclic voltammograms at 10 mV s^−1^ and the GCD curves at 0.5 A g^−1^, respectively, showing that the acidic medium, namely H_2_SO_4_, leads to superior capacitive performance, as indicated by higher specific current in the voltammogram and a longer discharge time.

The EIS Bode plots are depicted in Fig. 5(c and d). The impedance spectra obtained for the electrodes tested in H_2_SO_4_ and KOH were fitted using two CPEs. However, in Na_2_SO_4_, an additional Warburg element was required due to mass transport effects. The solution resistance was lower in H_2_SO_4_ (3.12 Ω cm^2^) compared to Na_2_SO_4_ (8.37 Ω cm^2^) and KOH (3.91 Ω cm^2^). Additionally, the higher CPE, power in H_2_SO_4_ (*n* = 0.87) relative to Na_2_SO_4_ (*n* = 0.65) and KOH (*n* = 0.60) reflects a response closer to ideal capacitive behaviour and greater charge storage capability in the acidic electrolyte.

The specific capacitance was determined from the discharge curves at 0.5 A g^−1^. The electrodes tested in 1 M H_2_SO_4_ electrolyte exhibited the highest specific capacitance, reaching 163.6 F g^−1^, followed by Na_2_SO_4_ (56.8 F g^−1^) and KOH (2.89 F g^−1^). Additionally, the composite exhibited a potential window of 0.725 V in H_2_SO_4_, higher than that in KOH and similar to that in Na_2_SO_4_. The superior capacitive performance observed in H_2_SO_4_ can be attributed to its high ionic conductivity and the efficient participation of H^+^ ions in the doping/dedoping process of the PPy chains, promoting fast, reversible faradaic redox reactions. Consequently, the electrode exhibits lower charge-transfer resistance, improved reaction kinetics, and a higher current response compared with the other electrolytes investigated.

These results confirmed that the acidic medium serves as the most suitable electrolyte for this specific composite, agreeing with existing literature.^25,26^ The acidic conditions help maintain PPy in its conductive protonated state, improving electronic conductivity throughout the polymer matrix. Combined with the excellent electrical conductivity of the spent graphite substrate, which provides efficient electron transport, the acidic electrolyte promotes favourable electron and ion transport, thereby enhancing the overall pseudocapacitive behaviour of the composite electrode. A comparison of the electrolyte-dependent performance of representative energy-storage systems is provided in Table S3.

### 3.5. Effect of the growth time

**Fig. 6:**
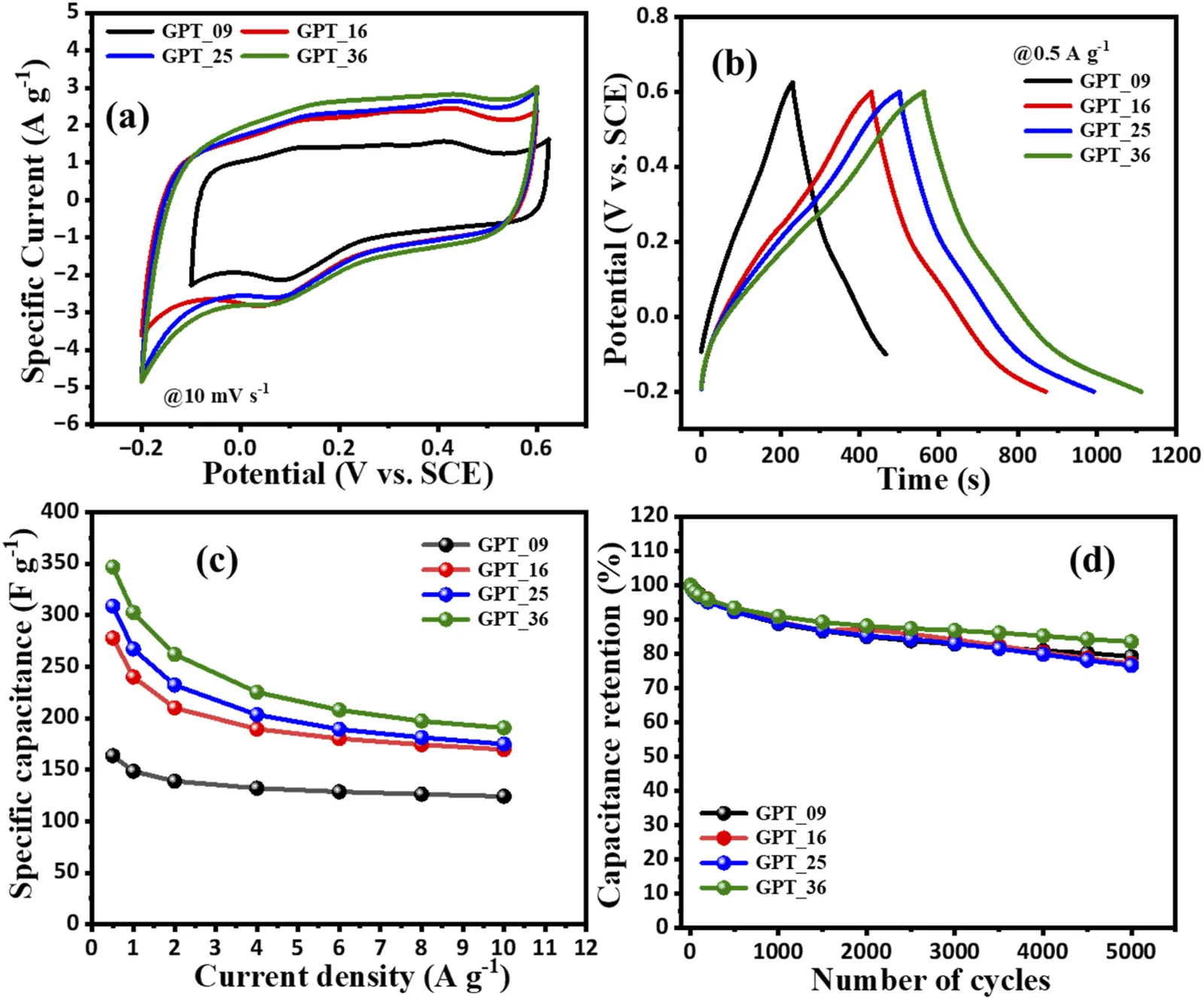
(a) CV curves at 10 mV s^−1^, (b) GCD curves at 0.5 A g^−1^, (c) specific capacitance as a function of the specific current, (d) capacitance retention variation for 5000 GCD cycles at 10 A g^−1^.

Fig. 6(a) compares the CV curves of the GPT_16, GPT_25, and GPT_36 electrodes at 10 mV s^−1^, in a potential window from −0.2 to 0.6 V *vs.* SCE in the 1 M H_2_SO_4_ electrolyte. The GPT-09 CV curve showed a working potential range of −0.1 to 0.625 V *vs.* SCE, which can be attributed to the response of the graphite that was not fully covered. All samples exhibited a coulombic efficiency close to 100%. The cyclic voltammograms of all samples exhibited a predominantly capacitive response, with minor redox features at approximately 0.4 V (*E*^a^_p_) and 0.0 V (*E*^c^_p_) *vs.* SCE. The overall charge-storage behaviour is therefore hybrid,^10^ combining a dominant non-faradaic electrical double-layer capacitance contribution from the graphite surface with an additional faradaic pseudocapacitive contribution from the PPy layer. The presence of redox peaks became more pronounced at lower scan rates because the extended timescale allows electrolyte ions to access a larger fraction of electroactive sites within the composite structure, facilitating faradaic charge-transfer reactions more than at higher rates, such as 100 mV s^−1^. Furthermore, the CV curve of GPT_36 exhibits a larger area compared to other samples because the PPy uniformly covers a denser region of the graphite layer. Increased PPy loading enhances electrochemical performance, as each unit mass participates more actively in charge transfer. Fig. 6(b) shows the GCD curves of GPT_09, GPT_16, GPT_25, and GPT_36 electrodes at 0.5 A g^−1^. Among all the samples, GPT_36 exhibits the longest charge/discharge time, confirming its superior energy storage capacity compared to the other composites. This behaviour is consistent with the CV results, where the GPT_36 voltammogram exhibits a larger area, further confirming its improved capacitive performance and efficient faradaic activity. The GCD curves of all samples exhibit a linear, reversible potential response, with minimal ohmic resistance and a dominant capacitive response. These features indicate an efficient energy-storage response governed by a pseudocapacitive mechanism.

Furthermore, Fig. 6(c) presents the specific capacitance of the different electrodes as a function of the applied current density. The specific capacitance of the GPT_09, GPT_16, GPT_25, and GPT_36 electrodes is 163.6, 277.7, 308.5, and 346.6 F g^−1^ at 0.5 A g^−1^, respectively. Among them, the electrode grown for a longer time – GPT_36 – exhibited the highest specific capacitance. Although increasing the deposition time promoted denser PPy particle growth and agglomeration, the sample deposited for 36 min exhibited the highest charge/discharge time, indicating that the increased amount of electroactive PPy outweighed any adverse effects associated with particle coarsening within the investigated deposition range. The larger polymer loading provides more redox-active sites, resulting in enhanced pseudocapacitive charge storage. Nevertheless, excessive deposition is expected to yield thicker, denser PPy films with reduced porosity, thereby limiting electrolyte penetration and increasing ion diffusion resistance. Consequently, deposition times beyond 36 min would likely lead to a decline in the electrochemical performance as the benefits of increased active material become offset by the poorer ion transport and reduced utilisation of the polymer.

Additionally, the cycling stability of the different electrodes was evaluated over 5000 GCD cycles at a specific current of 10 A g^−1^, as shown in Fig. 6(d). GPT_36 exhibited the highest stability, with capacitance retention of 83% after 5000 cycles, while the electrodes GPT_09, GPT_16, and GPT_25 displayed lower retentions of 79, 77, and 76%, respectively.

The role of the spent graphite as a PPy support was compared with commercial activated YP50 carbon, as shown in Fig. S4. The GPT_09 electrode exhibits more uniform PPy coverage than PPy@YP50 and a stronger electrochemical response, with a higher CV area and longer discharge time. At 0.5 A g^−1^, GPT_09 delivered a specific capacitance of 163.6 F g^−1^, compared with 139.5 F g^−1^ for PPy@YP50. However, PPy@YP50 showed superior cycling stability, reaching 103% capacitance retention after 5000 GCD cycles. Overall, these results demonstrate that recovered graphite can serve as an effective, more sustainable and inexpensive PPy support with the potential to replace commercial carbon. This confirms a new use for a large-scale and worldwide available battery-derived residue.

### 3.6. Effect of the growth potential

**Fig. 7:**
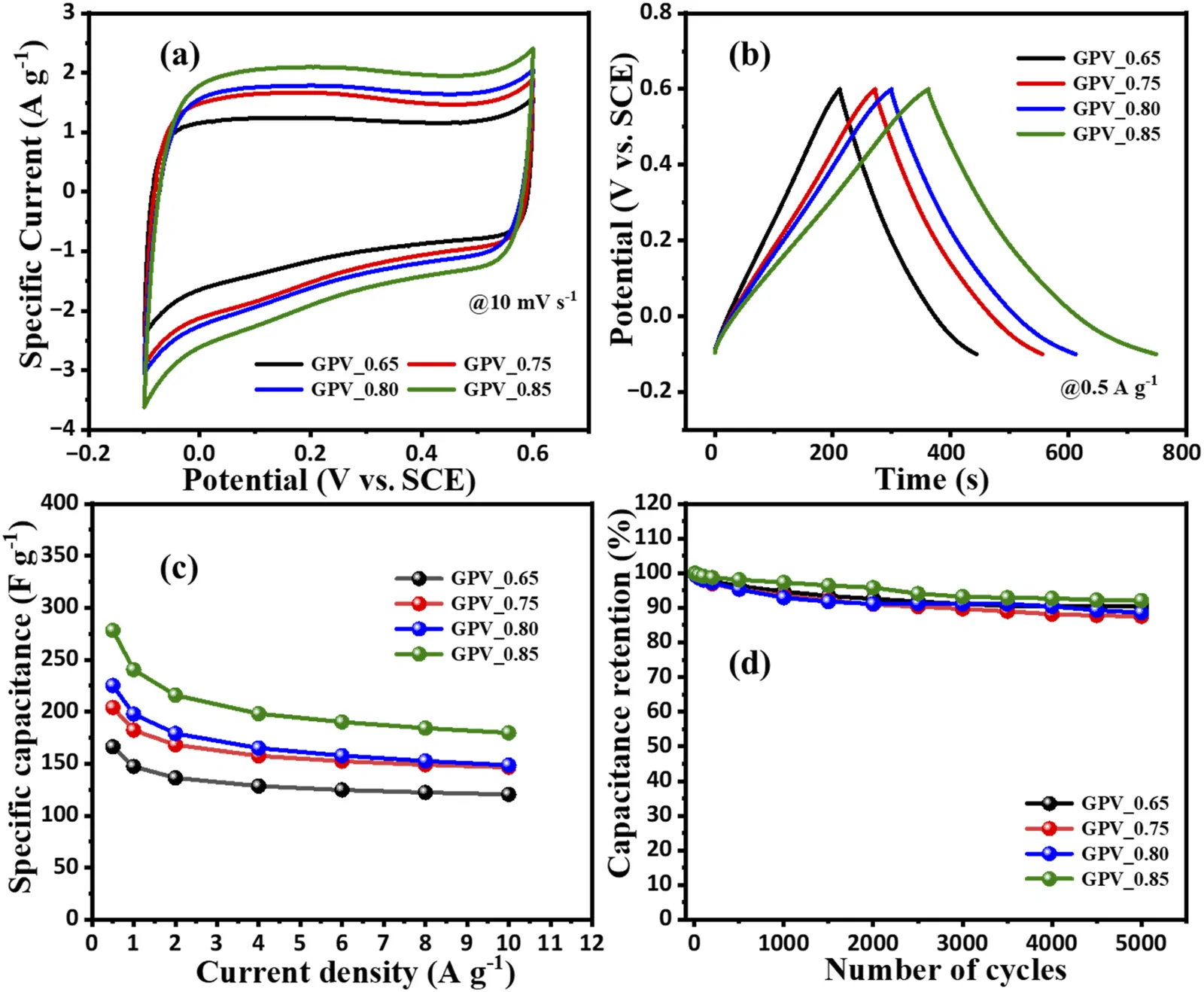
(a) CV curves at 10 mV s^−1^, (b) GCD curves at 0.5 A g^−1^, (c) specific capacitance as a function of the specific current, (d) capacitance retention variation for 5000 GCD cycles at 10 A g^−1^.

Fig. 7(a) compares the CV curves at 10 mV s^−1^ of GPV_0.65, GPV_0.75, GPV_0.80, and GPV_0.85 electrodes in a working potential window from −0.1 to 0.6 V *vs.* SCE measured in 1 M H_2_SO_4_. The cyclic voltammograms of all samples exhibit a rectangular shape with pseudocapacitive features. The modified electrodes exhibit higher specific currents as the growth potential increases, maintaining the same CV shape. Consequently, the GPV_0.85 electrode exhibits a voltammogram with a larger area than that of other samples due to the greater mass of PPy covering the graphite layer, as shown in Fig. 3(d). The higher PPy loading enhances electrochemical performance, as each mass unit participates more actively in charge transfer. Fig. 7(b) shows the GCD curves of the different electrodes at 0.5 A g^−1^. Among all the samples, GPV_0.85 presents the highest charge–discharge time, confirming its superior energy storage ability compared to the other composites. The charge–discharge behaviour exhibits low polarisation and a stable potential evolution, indicating efficient charge transfer and ion transport within the composite, reflecting a highly reversible charge–discharge process and a dominant capacitive charge storage. The longer discharge time obtained for GPV_0.85 indicates improved charge storage performance associated with increased polymer loading.

Furthermore, Fig. 7(c) presents the specific capacitance as a function of current density. The electrodes GPV_0.65, GPV_0.75, GPV_0.80, and GPV_0.85 displayed specific capacitance values at 0.5 A g^−1^ of 166.3, 204.1, 225.3, and 278.2 F g^−1^, respectively. As expected, GPV_0.85 exhibited the highest specific capacitance. The cycling stability of the different electrodes was assessed by 5000 GCD cycles at a current density of 10 A g^−1^. The modified electrodes exhibited a similar capacitance retention of about 90% after 5000 GCD cycles.

The similar capacitance–retention values indicate that increasing the deposition potential from 0.65 to 0.85 V did not markedly compromise cycling stability. The modest differences may arise from potential-dependent variations in PPy thickness, compactness, and interfacial adhesion, which influence mechanical stress during repeated doping/dedoping cycles.^19,37^ PPy expands and contracts during cycling, and thicker films accumulate more mechanical stress, leading to partial loss of electrical contact or degradation of electroactive sites. These results underscore the potential of this material for pseudocapacitive applications, making it well-suited for critical applications where stable high-power storage, reliability, and maintenance are key requirements.

### 3.7. EIS characterisation

**Fig. 8:**
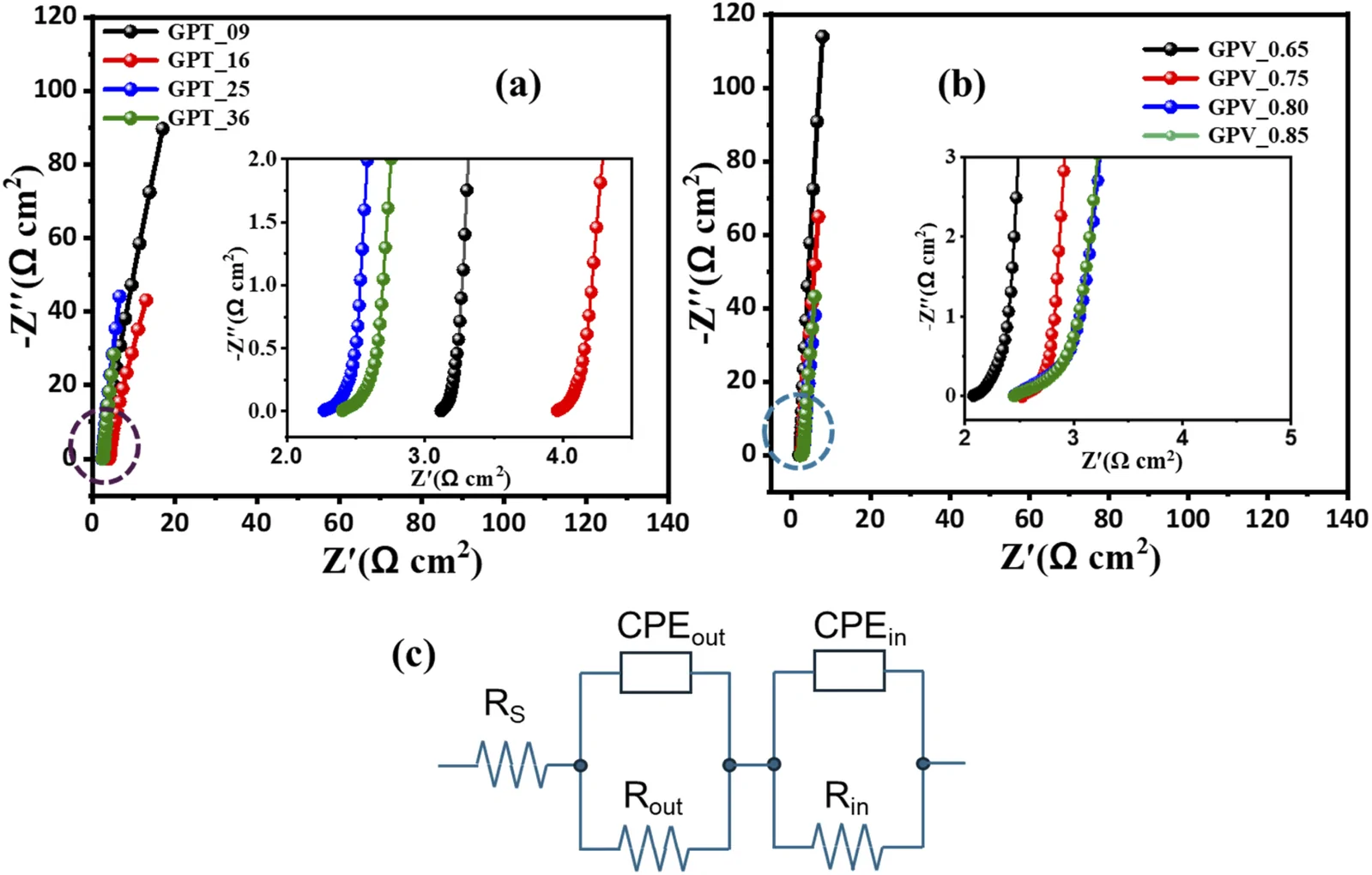
(a) Nyquist plots of GPT electrodes synthesised for different times (09, 16, 25 and 36 minutes) at 0.70 V, with the high frequency region highlighted in the inset (purple dotted circle); (b) Nyquist plots of GPV electrodes grown at different potentials (0.65, 0.75, 0.80 and 0.85 V) for 16 minutes, with the high frequency region highlighted in the inset (blue dotted circle); (c) electrical equivalent circuit used to fit the experimental data.

The EIS study provides a deeper understanding of the charge storage mechanisms in the electrode material. It reveals key parameters, including ion diffusion processes, charge-transfer kinetics, and the characteristics of the electrode–electrolyte interface. Fig. 8(a and b) show the Nyquist plots of the GPT and GPV electrodes, respectively. The impedance response is characterised by a non-ideal interfacial feature at intermediate frequencies, followed by a capacitive-dominated regime at low frequencies, indicative of efficient ion accommodation within the electrodes. The spectra show a pronounced semicircle at medium frequencies due to surface inhomogeneity, which leads to variations in capacitance and resistance, followed by a slightly tilted vertical line at low frequencies, characteristic of electrodes with a marked capacitive response. The phase angle plots, shown in Fig. S5, reveal the existence of two time constants at frequencies around 2500 and 0.01 Hz, respectively. The high-frequency time constant typically corresponds to the double layer or the interfacial charge transfer process that is characteristic of faster electrochemical mechanisms, while the lower frequency is related to slower mechanisms, including diffusion-controlled processes. The equivalent circuit shown in Fig. 8(c) was used to fit the EIS spectra. According to the data obtained, two resistive features were observed at high frequencies: the electrolyte resistance, *R*_s_, and an interfacial resistance, *R*_out_, at the electrode surface. The pseudocapacitive electrode response can be related to the presence of two different charge storage mechanisms. These mechanisms correspond to two constant phase elements. A faster electrostatic process at the electrode/electrolyte interface contributes to the double-layer capacitance at high frequencies (CPE_out_), parallel with an interfacial resistance (*R*_out_). In the low frequency region, slower processes take place due to faradaic charge storage in the inner layer, assigned to CPE_in_, and *R*_in_ is the resistance associated with those processes.

Tables 2 and 3 present the fitting parameters for all the composites. The electrolyte resistance ranges from 2.1 to 4 Ω cm^2^. This resistance primarily reflects the ionic resistance of the electrolyte, as well as the electrical resistance of the cell configuration and electrode contacts, rather than the intrinsic properties of the PPy layer. Since all measurements were performed under identical experimental conditions, the slight variations in *R*_s_ are most likely associated with normal experimental variability. The interfacial resistance (*R*_out_) of all composites is much lower compared to the inner one (*R*_in_), as expected. The polymeric matrix introduces additional ion-transport limitations within the active material, thereby increasing resistivity. Compared with the composites grown for different time pulses, as shown in Table 2, the outer resistance increases with increasing deposition time, which can promote the formation of higher branching of the polymeric chains, thereby hindering charge-carrier transport and, consequently, increasing the resistivity. For different growth potentials (Table 3), the inner resistance decreases as the applied potential increases. The higher potential also leads to more branched structures, which promote the formation of more porous films that facilitate the ionic diffusion into the polymeric matrix. The low chi-square values validate the use of the equivalent circuit to fit the experimental data.

**Table 2:** Summary of the parameters used for the fitting of the impedance spectra in Fig. 8(a)

*E*_g_ (V *vs.* SCE)	Time (min)	*R*_s_ (Ω cm^2^)	*Y*_out_ (Ω cm^−2^ s^*n*−1^)	*n*_out_	*R*_out_ (Ω cm^2^)	*Y*_in_ (Ω cm^−2^ s^*n*−1^)	*n*_in_	*R*_in_ (Ω cm^2^)	*χ* ^2^
0.70	9	3.12	0.08	0.87	0.06	0.15	0.95	907	2.30 × 10^−5^
0.70	16	3.97	0.14	0.73	0.10	0.28	0.92	426	2.31 × 10^−5^
0.70	25	2.29	0.50	0.56	0.20	0.32	0.97	1028	3.68 × 10^−6^
0.70	36	2.41	0.68	0.48	0.28	0.50	0.96	752	3.17 × 10^−6^

**Table 3:** Summary of the parameters used for the fitting of the impedance spectra in Fig. 8(b)

*E*_g_ (V *vs.* SCE)	Time (min)	*R*_s_ (Ω cm^2^)	*Y*_out_ (Ω cm^−2^ s^*n*−1^)	*n*_out_	*R*_out_ (Ω cm^2^)	*Y*_in_ (Ω cm^−2^ s^*n*−1)^	*n*_in_	*R*_in_ (Ω cm^2^)	*χ* ^2^
0.65	16	2.09	0.46	0.53	0.42	0.13	0.98	4072	5.08 × 10^−6^
0.75	16	2.51	0.42	0.48	0.32	0.22	0.97	2417	2.40 × 10^−6^
0.80	16	2.45	0.32	0.51	0.78	0.38	0.97	1266	1.89 × 10^−6^
0.85	16	2.89	0.55	0.44	0.74	0.27	0.97	1159	2.04 × 10^−6^

After the stability test, the electrodes were characterised by EIS. The respective fitting parameters are summarised in Tables 4 and 5. The impedance fitting indicates that long-term cycling has little effect on the ohmic resistance of the cell, confirming that the electrolyte and electrical contacts remain stable. The most significant ageing effect is the increase in both the interfacial resistance (*R*_out_) and, more prominently, the bulk charge-transfer resistance (*R*_in_). The increase in *R*_out_ suggests gradual modification of the PPy/electrolyte interface, likely associated with partial surface oxidation and a reduction in the number of electrochemically active sites. In contrast, the marked increase in *R*_in_ after cycling indicates degradation of the PPy matrix, resulting in reduced electronic conductivity and more hindered ion transport within the polymer. This behaviour is characteristic of conducting polymers undergoing long-term electrochemical ageing and is commonly attributed to partial overoxidation, disruption of the conjugated backbone, and structural rearrangement of the polymer network. Despite these changes, the nearly constant *R*_s_ and the high *n*_in_ values indicate that the electrolyte resistance remains unchanged and that the bulk capacitive behaviour of the electrode is largely maintained, even though charge transport becomes increasingly resistive.

**Table 4:** Summary of the parameters used for the fitting of the impedance spectra of the samples synthesised at different times after the stability test

*E*_g_ (V *vs.* SCE)	Time (min)	*R*_s_ (Ω cm^2^)	*Y*_out_ (Ω cm^−2^ s^*n*−1^)	*n*_out_	*R*_out_ (Ω cm^2^)	*Y*_in_ (Ω cm^−2^ s^*n*−1^)	*n*_in_	*R*_in_ (Ω cm^2^)	*χ* ^2^
0.70	9	3.05	0.63	0.56	0.12	0.15	0.98	1425	2.30 × 10^−5^
0.70	16	2.72	0.45	0.59	0.16	0.20	0.97	1585	2.31 × 10^−5^
0.70	25	2.22	0.60	0.45	0.25	0.21	0.97	1959	3.68 × 10^−6^
0.70	36	2.39	0.65	0.46	0.30	0.35	0.98	1367	3.17 × 10^−6^

**Table 5:** Summary of the parameters used for the fitting of the impedance spectra of the samples synthesised at different potentials after the stability test

*E*_g_ (V *vs.* SCE)	Time (min)	*R*_s_ (Ω cm^2^)	*Y*_out_ (Ω cm^−2^ s^*n*−1^)	*n*_out_	*R*_out_ (Ω cm^2^)	*Y*_in_ (Ω cm^−2^ s^*n*−1^)	*n*_in_	*R*_in_ (Ω cm^2^)	*χ* ^2^
0.65	16	2.06	0.36	0.55	0.41	0.09	0.99	4207	5.08 × 10^−6^
0.75	16	2.39	0.39	0.52	0.26	0.15	0.99	2993	2.40 × 10^−6^
0.80	16	2.36	0.26	0.55	0.53	0.27	0.98	1833	2.17 × 10^−6^
0.85	16	2.12	0.64	0.43	0.58	0.26	0.97	2038	3.87 × 10^−6^

Overall, these EIS results suggest that the dominant degradation mechanism during the stability test is intrinsic ageing of the PPy coating rather than deterioration of the electrolyte or current collector, which agrees well with the moderate loss in electrochemical performance expected after prolonged cycling.

### 3.8. Charge contribution analysis

The Trasatti method was employed to understand the structural variation of the electrodes by examining their charge contributions. The charge stored at the outer surface is influenced by the bulk diffusion coefficients, and it is readily accessible and governed by the “free” diffusion coefficient (*D*_f_). In contrast, the inner surface requires ions to move through the porous structure to reach the active sites, rendering this contribution less accessible and constrained by the effective diffusion coefficient (*D*_e_). *D*_e_ is influenced by the material's structural features, particularly its porosity and the complexity of its ion transport pathway, and is significantly lower than *D*_f_. To account for both inner and outer charge contributions, the electrode must be charged and discharged slowly to allow ions to diffuse internally. Consequently, cyclic voltammetry tests were carried out at very low scan rates (2, 4, 6, 8, and 10 mV s^−1^). Representative cyclic voltammograms recorded for GPT_09 at these scan rates are provided in Fig. S6.

Then, the overall charge stored is the sum of the inner and outer contributions (*q*_total_ = *q*_inner_ + *q*_outer_).

**Fig. 9:**
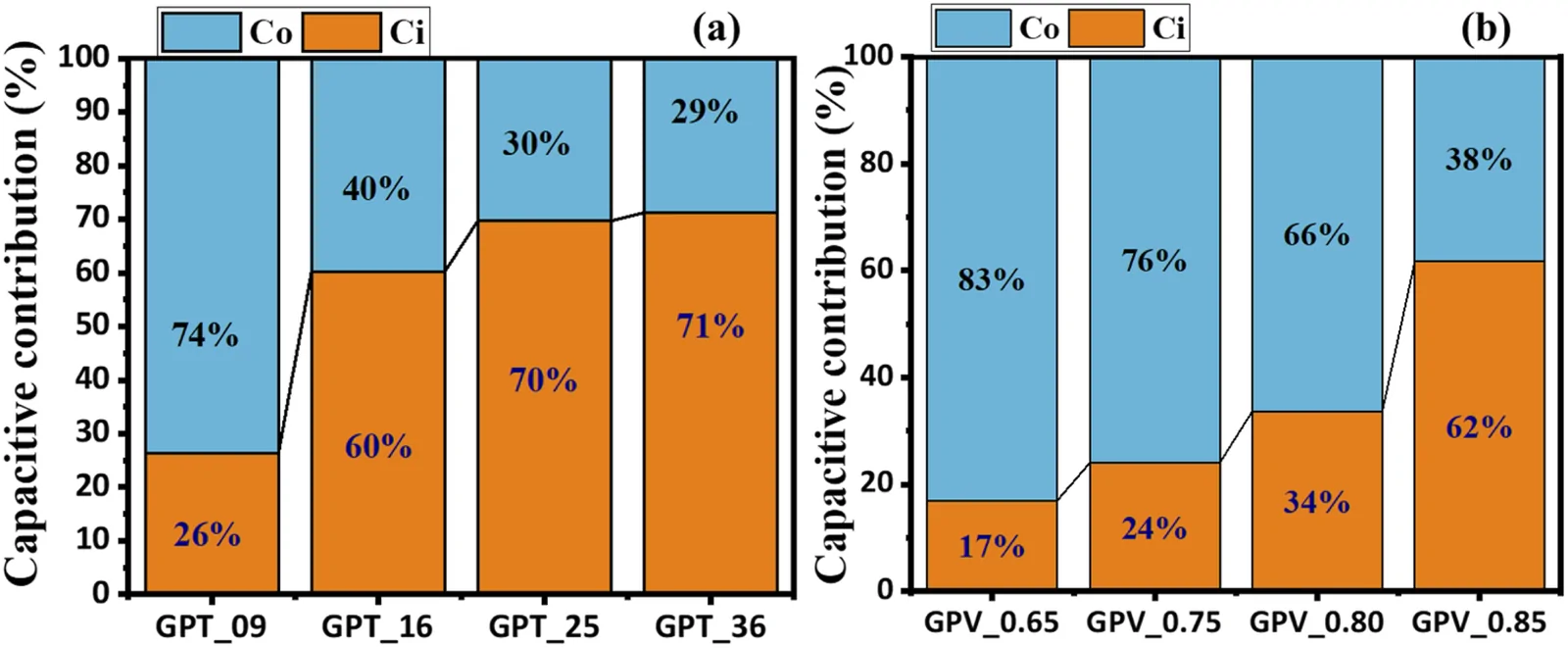
(a) Capacitive contribution analysis from the Trasatti method of GPT and GPV electrodes, *C*_i_ – inner capacitance, *C*_o_ – outer capacitance, (b) Capacitive contribution analysis from the Trasatti method, *C*_i_ – inner capacitance, *C*_o_ – outer capacitance.

Fig. 9 presents the charge contributions of the prepared composite materials. As shown in Fig. 9(a) and (b), the contribution of inner charges increases with the increasing growth time and potential, respectively. The modified electrodes GPT_09 and GPT_36 present 26% and 71% of inner contribution, respectively, while the modified electrodes GPV_0.65 and GPV_0.85 present inner contributions of 17% and 62%, respectively. This tendency is due to the film synthesised at a higher potential and for a longer time having greater thickness, a higher number of inner active sites, and, consequently, a higher inner contribution to charge storage. As the deposition time increases, the PPy film becomes thicker, creating additional electroactive sites within the polymer bulk that contribute to diffusion-controlled charge storage, thereby increasing *C*_i_.^19^ In addition, increasing the deposition potential accelerates polymer growth, resulting in thicker PPy layers with a greater fraction of electroactive sites located within the film. Consequently, ion diffusion into the polymer becomes more significant, increasing the contribution of the inner capacitance. Overall, GPV_0.85 exhibited the best electrochemical performance among the electrodes prepared at a fixed deposition time of 16 min, combining high capacitance with a substantial contribution from inner charge storage. Therefore, this electrode was selected for symmetric cell assembly.

### 3.9. Symmetric cell characterisation

Two identical GPV_0.85 mass-loaded electrodes were assembled in a symmetric cell and evaluated in a two-electrode configuration using 1 M H_2_SO_4_ electrolyte. For the two-electrode cell, the energy density (*E*) was calculated according to eqn (5) and the power density (*P*) according to eqn (6), respectively.5
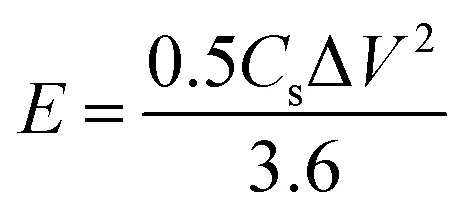
6
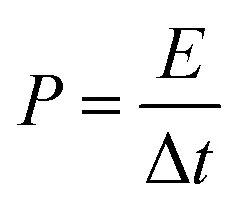


**Fig. 10:**
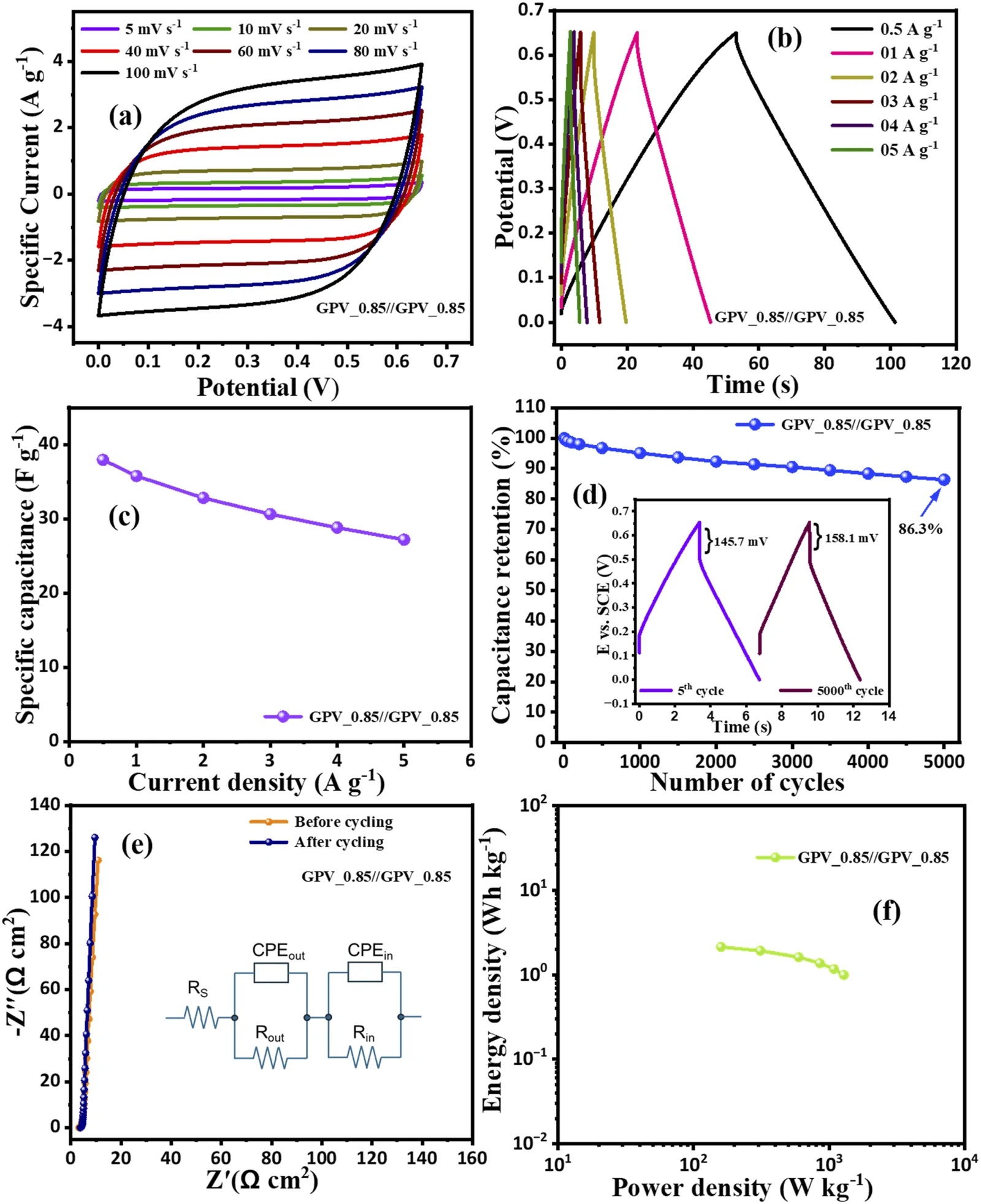
(a) CV curves at different scan rates, (b) GCD curves at different current densities, (c) specific capacitance as a function of the current density, (d) capacitance retention variation for 5000 GCD cycles at 5 A g^−1^ (inset displays the charge discharge of 5th and 5000th cycle highlighting the increase in ohmic drop), (e) Nyquist plot (inset of equivalent circuit), (f) Ragone plot displaying the energy density as a function of the power density.

Fig. 10(a) presents the cyclic voltammograms at various scan rates, which exhibit a similar shape to single electrodes, while the GCD profiles obtained at different applied currents display a stable and reversible potential response, confirming the good electrochemical behaviour, depicted in Fig. 10(b), within the potential window of 700 mV. Fig. 10(c) presents the specific capacitance as a function of the current density, with a specific capacitance of 37.8 F g^−1^ at 0.5 A g^−1^, which is lower than that obtained in the three-electrode configuration because the measured response includes the contribution of both electrodes and the full cell resistance. The reduction in capacitance observed in the two-electrode configuration compared to the three-electrode system is expected, as the device response includes contributions from both electrodes and increased internal resistance, providing a more realistic evaluation of practical performance. Fig. 10(d) shows the evolution of the capacitance retention after 5000 GCD cycles at 5 A g^−1^. The device exhibited 86.3% capacitance retention after long cycling, accompanied by a slight increase in the ohmic drop from 146 to 158 mV, as shown in the inset of Fig. 10(d).

The Nyquist plot obtained from the EIS test is shown in Fig. 10(e). The impedance spectra were analysed and fitted using the equivalent circuit employed for the half-cell system. The Nyquist plot exhibited highly depressed semicircles in the high-frequency region, and the solution resistance increased from 3.1 to 3.6 Ω cm^2^ before and after the stability tests, which can be attributed to slight electrode degradation or cell ageing. The capacitance was calculated from the fitting parameters and varied from 233 to 206 mF cm^−2^, before and after the stability test. The rise in the imaginary impedance at 10 mHz following the long cycling indicates a reduction in the device's capacitance (*Z*″ = 1/*jwc*). EIS can also provide insights into additional parameters of energy-storage electrodes, such as ion transport and pore accessibility, through the complex capacitance analysis, which includes the real and imaginary components of the capacitance, as illustrated in Fig. S7. This analysis is expressed by the following equations.

7 *C*(*ω*) = *C*′(*ω*) − *jC*″(*ω*)

where8
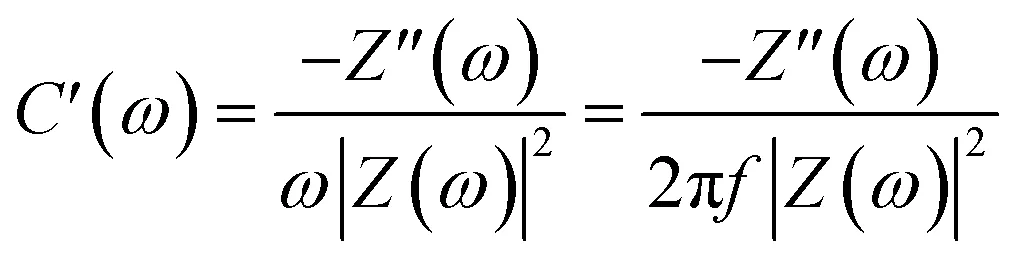
9
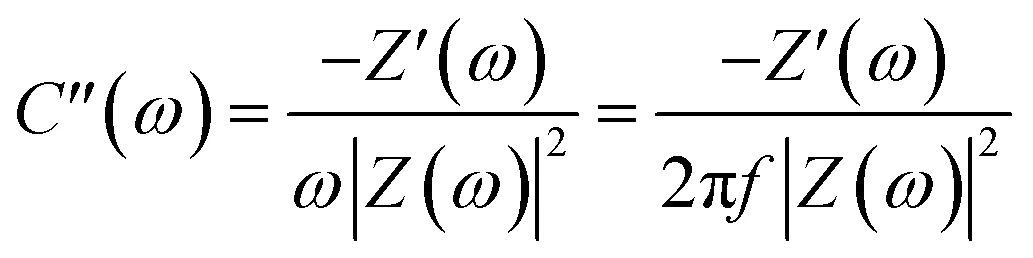
and

10 *Z*(*ω*) = *Z*′(*ω*) + *Z*″(*ω*)

where *ω* is the angular frequency, *C*′(*ω*) is the real capacitance, and *C*″(*ω*) is the imaginary capacitance, respectively; *C*(*ω*) is a complex capacitance, *Z*′(*ω*) is the real part of the impedance and *Z*″(*ω*) is the imaginary part of the impedance, respectively.^45^ Fig. S7 illustrates the fluctuation of *C*′(*ω*) and *C*″(*ω*) as a function of frequency. *C*′(*ω*) shows poor capacitive behaviour at high frequency, as shown in Fig. S7, because ions can only access the external surface within a short timescale. As the frequency decreases, *C*′(*ω*) increases sharply, reflecting improved ion penetration into the internal pore network. The imaginary capacitance *C*″(*ω*) shows a clear maximum at a specific frequency, denoted as *f*_0_. This frequency can be associated with the dielectric relaxation time. The supercapacitor's merit factor is given by the equation *t*_0_ = 1/*f*_0_. The relaxation time of 3.98 seconds represents the minimum duration required to discharge the entire energy of the device with an efficiency higher than 50%. A thorough understanding of these parameters can aid in optimising the performance of high-power electrodes for various practical applications. These findings suggest that the novel composite material has promising characteristics for high-power energy storage applications.

The Ragone plot, shown in Fig. 10(f), shows that the cell has a maximum power density of 1.2 kW kg^−1^ at an energy density of 0.9 Wh kg^−1^. Although the energy density is modest, the device combines appreciable power output with stable cycling while directly valorising graphite recovered from spent lithium-ion batteries. Vitto *et al.*^46^ also investigated the use of a waste graphite electrode and obtained 20.1 F g^−1^ at 0.1 A g^−1^. Chin Chung Lai successfully fabricated a symmetric carbon-based electrode that reached 30.10 F g^−1^ at 0.5 A g^−1^.^47^ Among asymmetric configurations, Aneesh Anand reported a Na_2/3_MnO_2_‖AC device that exhibits a specific capacitance of 37.7 F g^−1^ at 0.1 A g^−1^.^48^ These values are not directly comparable because of differences in electrode composition, cell configuration, current density, and capacitance normalisation. Nevertheless, the present work demonstrates that recovered LIB graphite can be directly upgraded through PPy electropolymerisation to provide stable high-power electrochemical performance without requiring graphene conversion or high-temperature activation. A broader comparison with representative PPy-based energy-storage systems is provided in Table 6.

**Table 6:** Comparison of representative PPy-based energy-storage systems with the present spent-graphite/PPy device

Electrode system	Test configuration	Key performance	Critical comparison	Ref.
Graphite/GO/PPy	1 M H_2_SO_4_; electrode-level	1320 mF cm^−2^; 83% retention after 5000 cycles	High response, but requires GO-modified commercial graphite	25
rGO/PPy	1 M H_2_SO_4_; electrode-level	424 F g^−1^ at 1 A g^−1^	Strong capacitance, but relies on engineered rGO scaffold	26
rGO-MnO_2_-PPy	1 M H_2_SO_4_; symmetric two-electrode cell	220.16 F g^−1^ at 0.5 A g^−1^; 86.08% after 5000 cycles	Enhanced by additional MnO_2_ pseudocapacitive phase	49
Electro-grafted PPy/carbon cloth	PVA/H_2_SO_4_ gel electrolyte	1212.5 mF cm^−2^; device: 333 mF cm^−2^; 74% after 5000 cycles	High capacitance, but lower device-level cycling stability	50
Spent graphite/PPy, this work	1 M H_2_SO_4_; symmetric cell	37.8 F g^−1^ at 0.5 A g^−1^; 86.3% retention after 5000 cycles	Directly upgrades recovered LIB graphite without GO/rGO conversion	This work

It is also noteworthy that most reported systems rely on commercial graphite, GO/rGO, multistep processing or additional metal oxides. In contrast, the present approach directly electropolymerises PPy onto recovered LIB graphite and demonstrates its potential as an electrode in a symmetric cell. Although the reported values are not directly comparable because of differences in test configuration and normalisation, the results demonstrate a simple route for the valorisation of an abundant graphite-rich battery waste.

## 4. Conclusions

This study demonstrates the reuse of spent graphite for energy-storage applications by functionalising it with polypyrrole through direct electropolymerisation, thereby transforming a low-value graphite-rich recycling residue into an electroactive pseudocapacitive material. Electropolymerisation offers an efficient, cost-effective, and scalable approach to improve the surface activity and charge transport of spent graphite. PPy was selected for its high electrical conductivity, pseudocapacitive behaviour, and ease of electropolymerisation for electrode functionalisation, thereby forming a uniform polymer coating. This study examines the effects of different times and potentials on the growth of PPy on graphite in a 1 M H_2_SO_4_ electrolyte. With increased time and applied potential during polymerisation, the electrode's specific capacitance increases. The GPV_0.85 electrode, which delivered the best electrochemical performance among the samples prepared with a 16 min deposition time, was selected for symmetric cell assembly. The resulting GPV_0.85//GPV_0.85 device achieved a specific capacitance of 37.8 F g^−1^ at 0.5 A g^−1^, high-rate capability, and long-term cyclic stability (86.3%) after 5000 cycles at 5 A g^−1^. The cell achieved a maximum power density of 1200 W kg^−1^ at an energy density of 0.9 Wh kg^−1^. This study provides insight into the functionalisation of spent graphite to enhance its electrochemical response as a pseudocapacitive electrode material. It demonstrates that spent graphite can be directly upgraded into an electroactive scaffold *via* simple electropolymerisation, achieving competitive capacitive performance and enabling its use as a sustainable alternative to commercial carbon materials for high-power electrochemical electrodes.

## Conflicts of interest

There are no conflicts to declare.

## Supplementary Material

RA-OLF-D6RA05302E-s001

## Data Availability

The data supporting the findings of this study are available within the article and its supplementary information (SI). All experimental data generated and analysed during this study are included in the manuscript and the accompanying SI file. Supplementary information is available. See DOI: https://doi.org/10.1039/d6ra05302e. References cited in the SI are listed separately within the SI file.
