# Supplementary material for: Reusing spent graphite through direct electropolymerisation of polypyrrole for sustainable pseudocapacitive electrodes
Source: RSC Adv. 2026 Aug 20;16(41):49112–26. doi: 10.1039/d6ra05302e (PMC13491317; doi:10.1039/d6ra05302e)
Supplement: Supplementary file 1 [file RA-016-D6RA05302E-s001.pdf]

Supplementary Information (SI) for RSC Advances.
This journal is © The Royal Society of Chemistry 2026

# Supplementary file

## Reusing Spent Graphite through Direct Electropolymerisation of Polypyrrole for Sustainable Pseudocapacitive Electrodes.

Nishat S. Khan[1], Maryna Taryba[1], R. G. Duarte[2], M. F. Montemor[1,] R. S. Sampaio[1].

[1]CQE - Centro de Química Estrutural, IMS - Institute of Molecular Sciences, Departamento de Engenharia Química, Instituto Superior Técnico, Universidade de Lisboa, Av. Rovisco Pais, 1049-001 Lisboa

[2]Instituto Politécnico de Setúbal, EST Barreiro, 2839-001 Lavradio, Portugal.

*Table S1: Gravimetrically determined PPy mass of the composite electrodes.*

| Potential (V vs SCE) | Time (minutes) | Mass of PPy (mg) |
|---|---|---|
| 0.70 | 9 | 0.78 |
| 0.70 | 16 | 1.68 |
| 0.70 | 25 | 1.83 |
| 0.70 | 36 | 2.59 |
| 0.65 | 16 | 0.78 |
| 0.75 | 16 | 1.05 |
| 0.80 | 16 | 1.63 |
| 0.85 | 16 | 1.64 |

*Table S2: Elemental composition of the PPy-coated graphite electrodes.*

| Element name | C (wt%) | N (wt%) | O (wt%) | Al (wt%) | Cl (wt%) | Mn (wt%) | Co (wt%) | Ni (wt%) |
|---|---|---|---|---|---|---|---|---|
| **GPT_09** | 51.500 | 8.900 | 18.800 | 5.300 | 14.700 | 0.200 | 0.300 | 0.300 |
| **GPT_16** | 39.740 | 9.409 | 21.922 | 1.802 | 26.527 | 0.100 | 0.200 | 0.300 |
| **GPT_25** | 58.258 | 7.508 | 4.404 | 1.101 | 28.028 | 0.100 | 0.300 | 0.300 |
| **GPT_36** | 35.800 | 10.000 | 24.000 | 0.900 | 28.800 | 0.000 | 0.200 | 0.300 |
| **GPV_0.65** | 61.277 | 7.485 | 15.769 | 5.389 | 9.182 | 0.200 | 0.399 | 0.299 |
| **GPV_0.75** | 48.700 | 10.100 | 21.100 | 2.100 | 17.400 | 0.100 | 0.200 | 0.300 |

| | | | | | | | | |
|---|---|---|---|---|---|---|---|---|
| **GPV_0.80** | 48.500 | 9.300 | 20.800 | 4.000 | 16.300 | 0.500 | 0.300 | 0.300 |
| **GPV_0.85** | 49.000 | 9.500 | 20.300 | 4.500 | 15.900 | 0.100 | 0.300 | 0.400 |

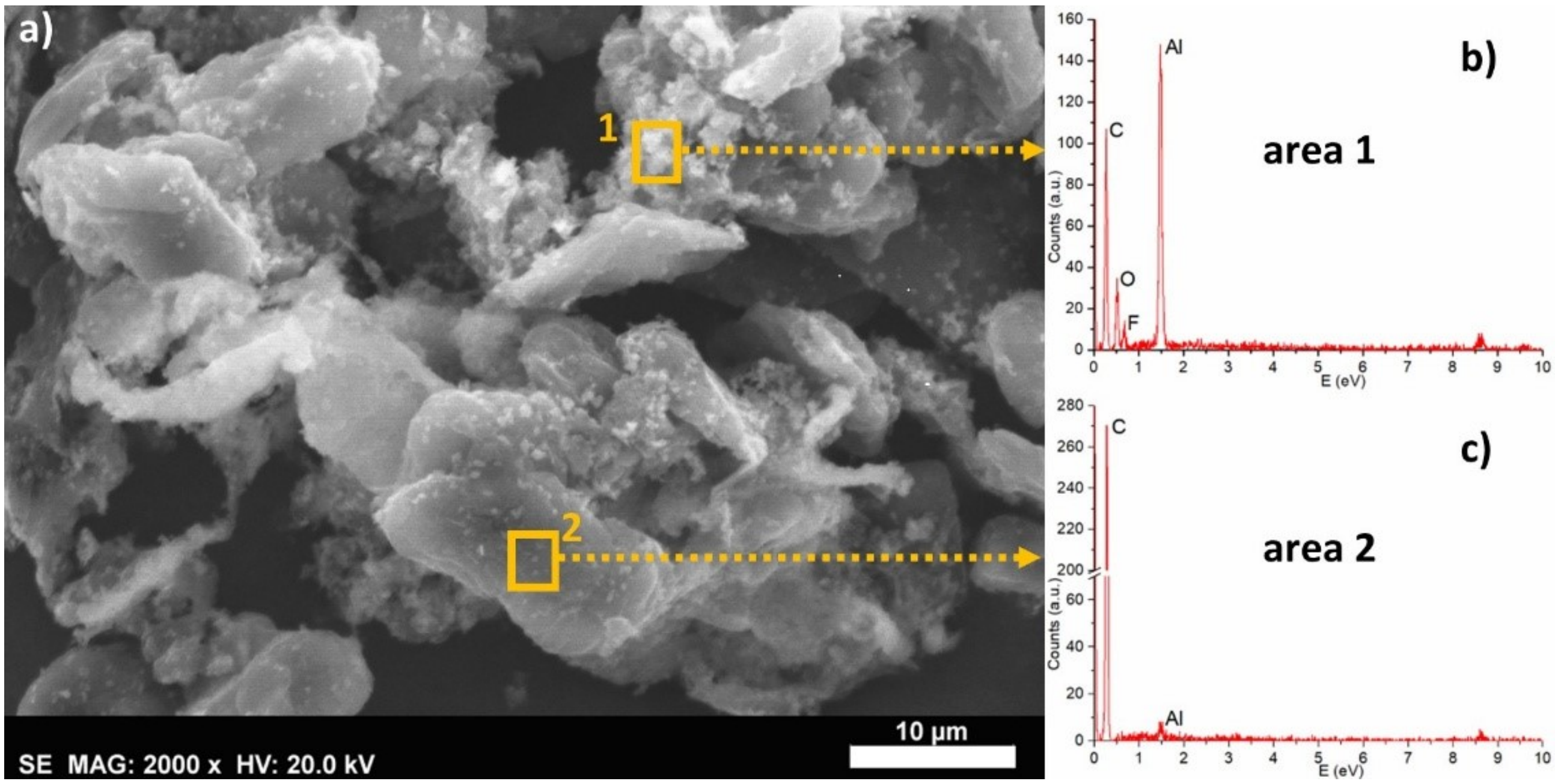


*Figure S1: (a) SEM micrographs of spent graphite/LBM; (b-c) corresponding EDS spectra acquired from the selected regions.*

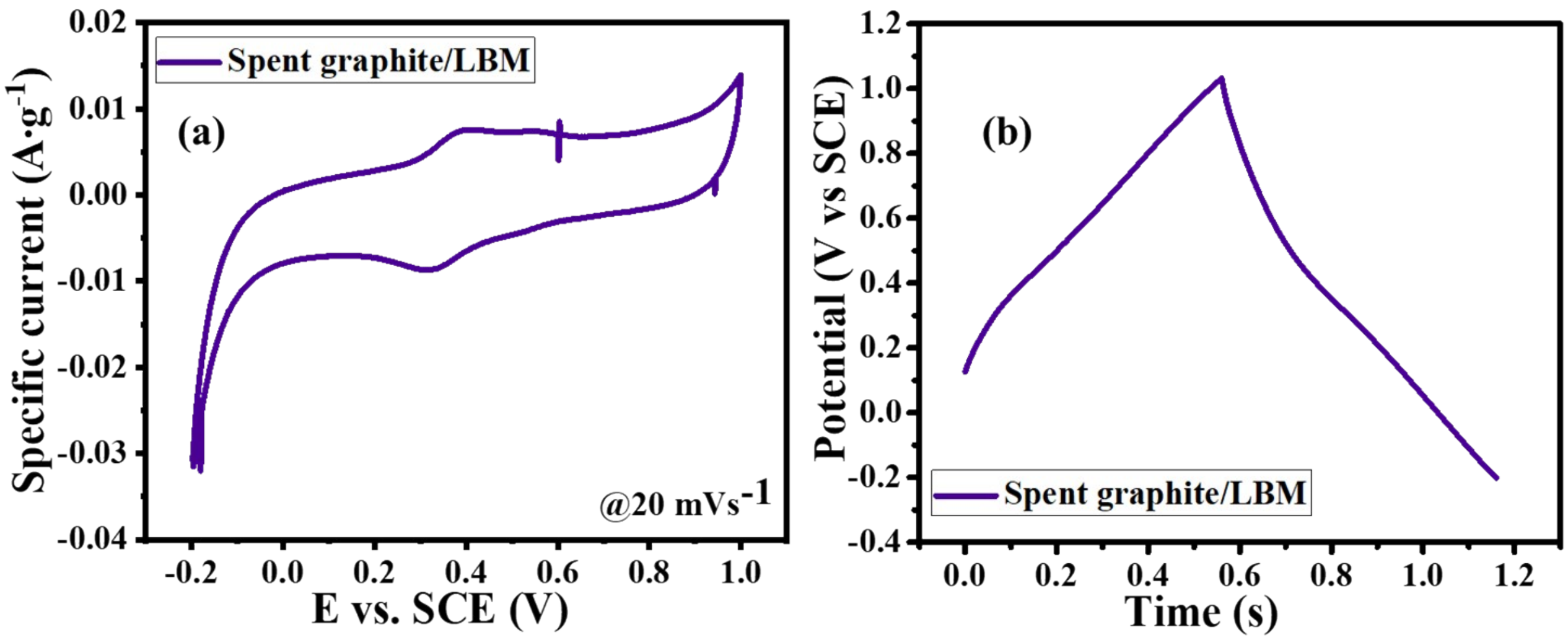


*Figure S2: Electrochemical study of spent graphite: (a) CV curves recorded at a scan rate of 20 mV $s^{-1}$; (b) GCD curves measured at a current density of 0.5 A $g^{-1}$ in 1 M $H_2SO_4$.*

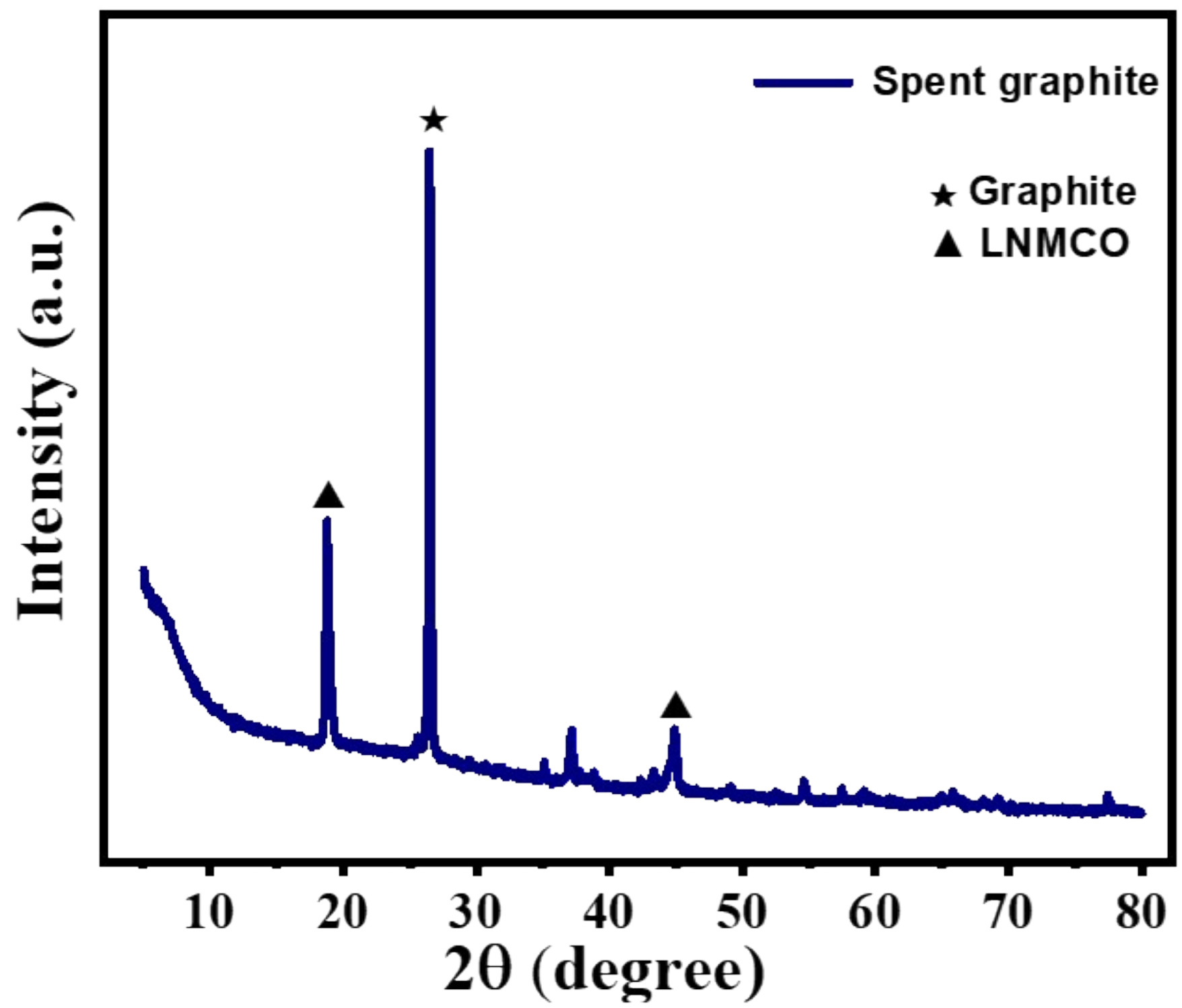


*Figure S3: X-ray diffraction pattern of graphite recovered from spent lithium-ion batteries.*

*Table S3: Influence of electrolyte chemistry and configuration on the electrochemical performance of representative supercapacitor systems.*

| Electrode/material | Electrolyte and configuration | Key performance | Electrolyte effect | Ref |
|---|---|---|---|---|
| PPy-MO nanostructures | 0.2 M HCl; three-electrode | 1914 F $g^{-1}$ at 10 mV $s^{-1}$ | Acidic conditions enhanced PPy redox activity. | 1 |
| $CuNi_2O_3$@N-OCCFs//N-OCCFs | PVA-1 M NaCl nanofibre electrolyte; asymmetric flexible cell, 2.2 V | 94.6 F $g^{-1}$; 63.6 Wh $kg^{-1}$ at 1100.6 W $kg^{-1}$ | The nanofibre electrolyte extended the stable voltage window. | 2 |
| $SrWO_4$/rGO@NF//Sr $WO_4$/rGO@NF | PVA-KOH gel; symmetric cell, 1.6 V | 83.6 F $g^{-1}$; 26.13 Wh $kg^{-1}$ at 2159.5 W $kg^{-1}$ | KOH-supported reversible tungstate redox reactions. | 3 |

| | | | | |
|---|---|---|---|---|
| AC//CN/$Gd_2S_3$ | 2 M KOH; solid-state asymmetric cell | 70.95 Wh kg$^{-1}$ at 250 W kg$^{-1}$ | KOH promoted utilisation of the redox-active sulfide electrode. | [4] |
| NW-3@GO//NW-3@GO | 1 M KOH; solid-state symmetric cell | 132.8 Wh kg$^{-1}$ at 2863 W kg$^{-1}$; 85% retention after 5000 cycles | The alkaline electrolyte enabled stable charge storage. | [5] |
| PPy/graphite | 1.2 M KCl; 1.0 V | 324.5 F g$^{-1}$ at 1 A g$^{-1}$ | The neutral electrolyte maintained stable PPy electroactivity. | [6] |
| Spent graphite/PPy, this work | 1 M $H_2SO_4$; three-electrode, 0.725 V | 163.6 F g$^{-1}$ at 0.5 A g$^{-1}$ | Rapid proton transport favoured PPy doping/dedoping. | This work |
| Spent graphite/PPy, this work | 1 M $Na_2SO_4$; three-electrode, 0.75 V | 56.8 F g$^{-1}$ at 0.5 A g$^{-1}$ | Higher resistance limited ion transport and active-site utilisation. | This work |
| Spent graphite/PPy, this work | 1 M KOH; three-electrode, 0.60 V | 2.89 F g$^{-1}$ at 0.5 A g$^{-1}$ | Alkaline deprotonation suppressed PPy conductivity and redox activity. | This work |

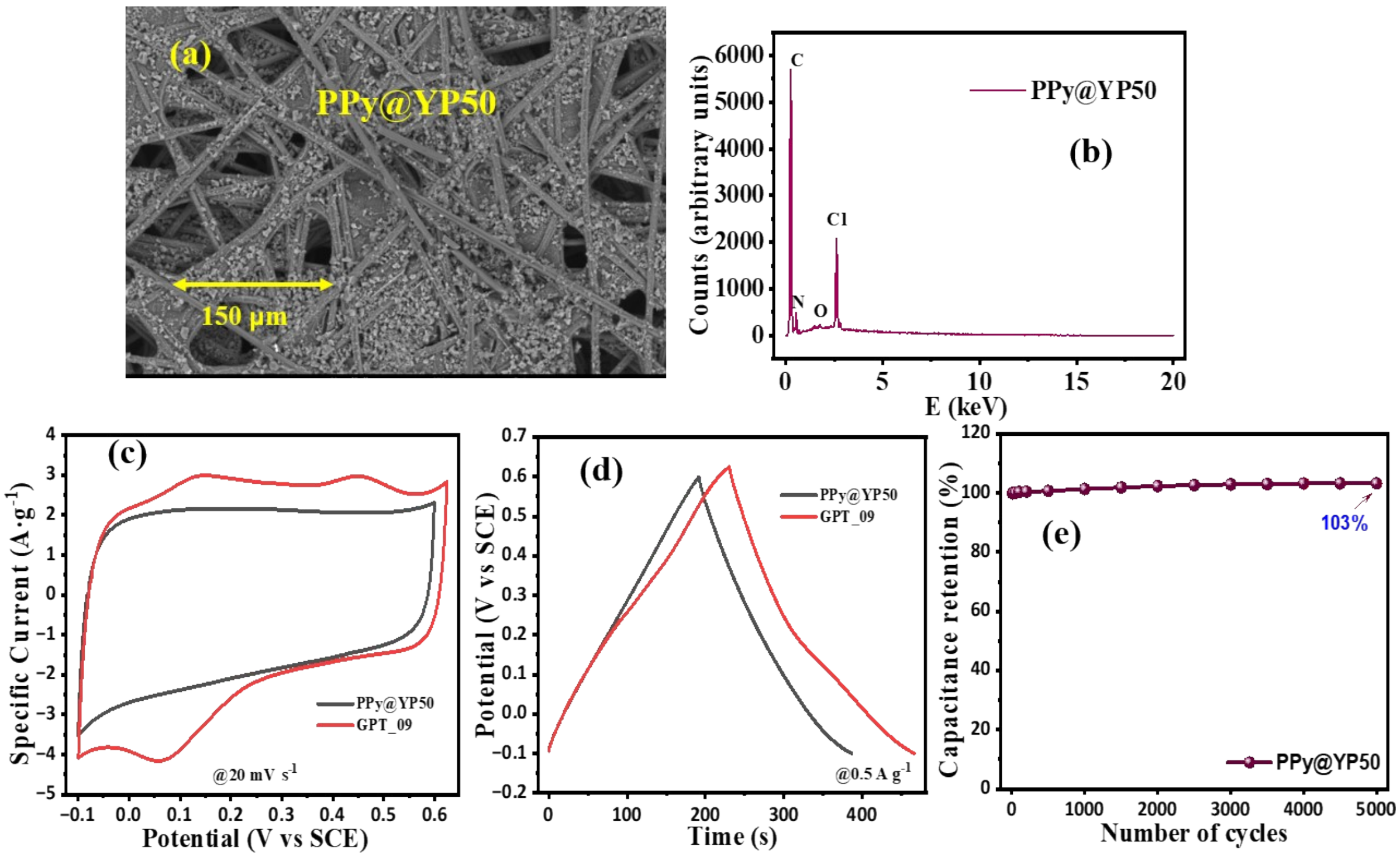


*Figure S4: Comparison of PPy@YP50 and GPT_09: (a) SEM image and (b) EDS spectrum of PPy@YP50; (c) CV curves at 20 mV $s^{-1}$; (d) GCD profiles at 0.5 A $g^{-1}$ and (e) capacitance retention of PPy@YP50 over 5000 GCD cycles.*

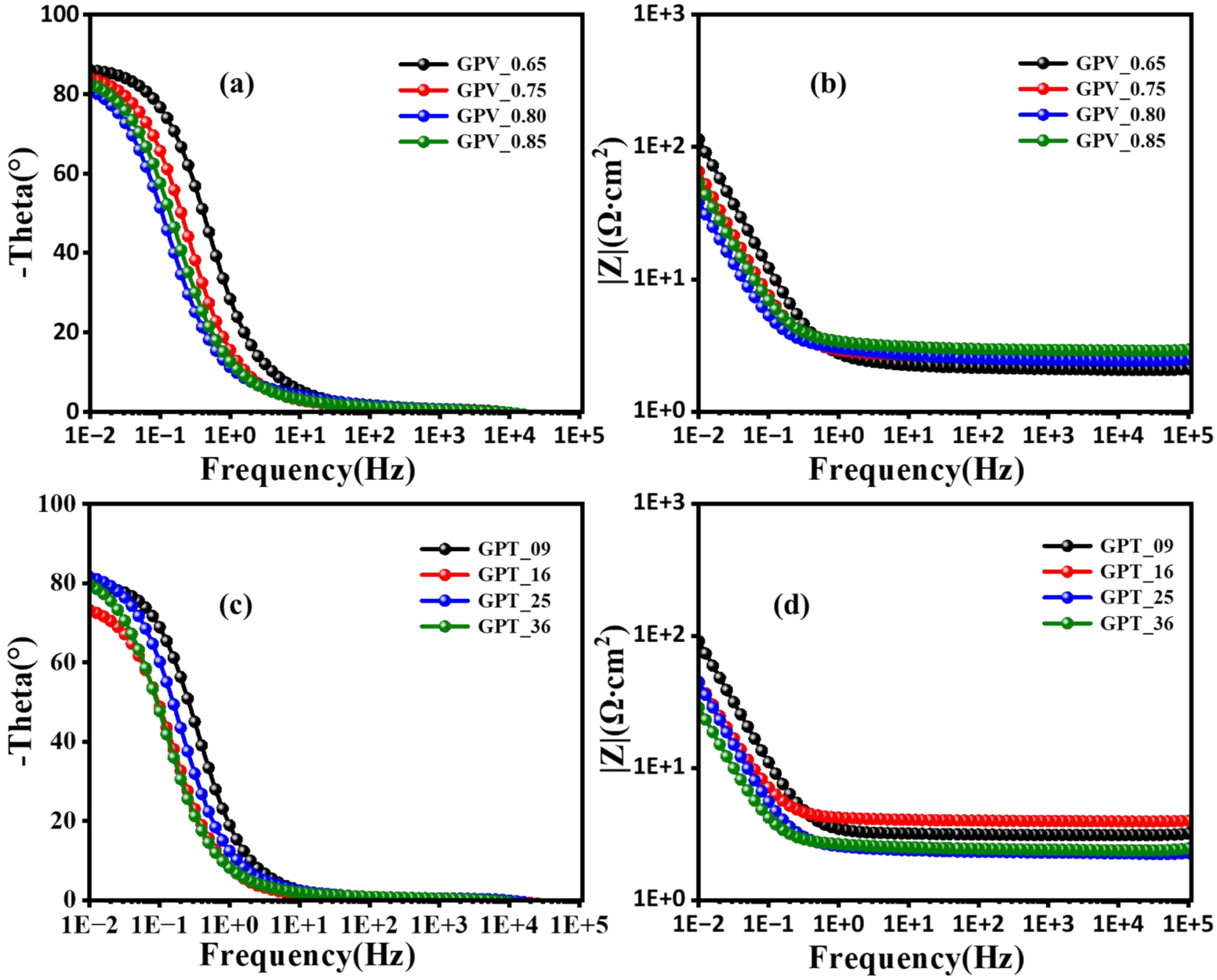


*Figure S5: Bode plots of (a-b) GPV and (c-d) GPT electrodes.*

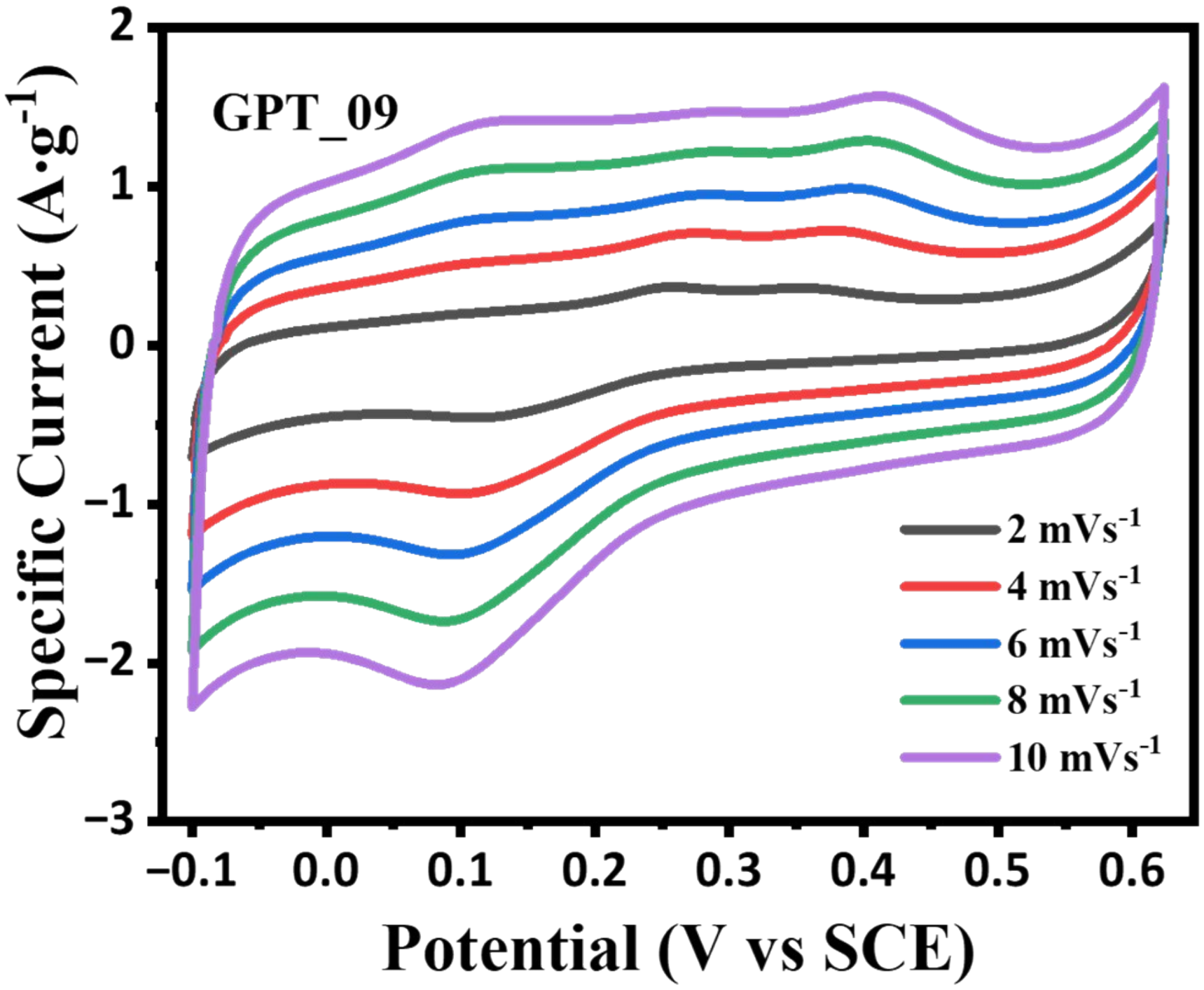


*Figure S6: Representative cyclic voltammograms of GPT_09 recorded at scan rates of 2, 4, 6, 8 and 10 mV s⁻¹ in 1 M $H_2SO_4$.*

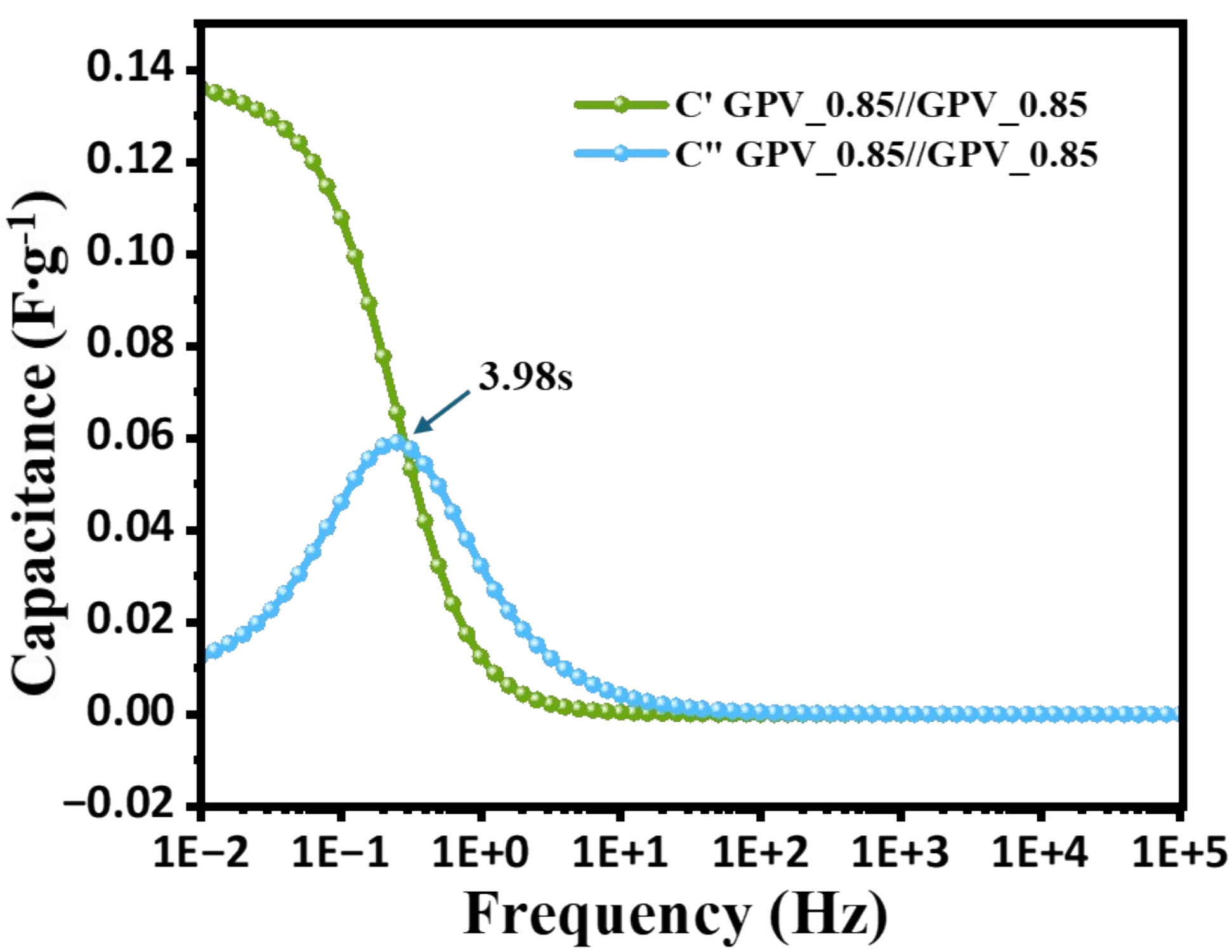


*Figure S7: Complex capacitance analysis.*

References

1 D. V. Tumacder, I. M. Minisy, O. Taboubi and P. Bober, Polymers, 2023, 15, 4140.

2 P. S. Kumar, J. Bae, J. W. Roh, Y. Min and S. Lee, Nano Converg., 2025, 12, 21.

3 S. Ramar, S. K. Ponnaiah, B. K. Raja, M. Govindaraj and A. J. Selvi, *Mater. Adv.*, 2026, **7**, 5647–5661.

4 P. Sathish Kumar, Y. Min, D. C. Hyun, J. H. Choi and S. Lee, *J. Energy Storage*, 2023, **74**, 109385.

5 S. Ramar, P. Sathish Kumar, M. Govindaraj, M. K. Muthukumaran, B. K. Raja and J. Arockia Selvi, *Energy Fuels*, 2025, **39**, 10070–10082.

6 D. Pham, R. Gouafong, J. Irvin, B. McKinney, J. Dileonardi, M. Cox and A. Aphale, Sci. Rep., 2026, 16, 11720.
